# Characteristics of patients with undiagnosed stage 3 chronic kidney disease: results from an observational study (REVEAL-CKD) in China

**DOI:** 10.1016/j.lanwpc.2024.101275

**Published:** 2025-01-04

**Authors:** Sheng Nie, Shiyu Zhou, Claudia Cabrera, Shan Chen, Mengyun Jia, Shiyu Zhang, Licong Su, Qi Gao, Navdeep Tangri, Fan Fan Hou

**Affiliations:** a Division of Nephrology, National Clinical Research Centre for Kidney Disease, State Key Laboratory of Organ Failure Research, Nanfang Hospital, Southern Medical University, Guangzhou, China; b Real World Science and Analytics, Evidence, BioPharmaceutical Medical, AstraZeneca, Mölndal, Sweden; c Medical Affairs CVRM, AstraZeneca Pharmaceutical (Hangzhou) Co. Ltd. Company, Hangzhou, China; d Biometrics and RWE, Evidence Generation, AstraZeneca Investment (CN) Co Ltd, Shanghai, China; e Department of Internal Medicine, University of Manitoba, Winnipeg, Manitoba, Canada

**Keywords:** Chronic kidney disease, Early stage, Diagnosis, Management, China

## Abstract

**Background:**

Early diagnosis of chronic kidney disease (CKD) is crucial for timely intervention to delay disease progression and improve patient outcomes. However, data for clinical characteristics of Chinese patients with undiagnosed, early-stage CKD are lacking.

**Methods:**

REVEAL-CKD is a multinational, observational study using real-world data in selected countries to describe factors associated with undiagnosed stage 3 CKD, time to diagnosis, and CKD management post diagnosis. We analysed patient data from 20 hospitals in the China Renal Data System. Adult patients with two consecutive estimated glomerular filtration rate (eGFR) measurements indicating stage 3 CKD (30–<60 ml/min/1.73 m^2^) recorded >90–730 days apart from 2015 to 2020 were eligible.

**Findings:**

Among 35,222 eligible patients, 25,214 (71.6%) were undiagnosed (lacked a CKD diagnostic code before and up to six months post-second-qualifying-eGFR). Only 2344 (9.3%) undiagnosed patients eventually received a delayed diagnosis, whose median time to diagnosis was 18.1 (95% CI: 17.6–18.8) months. Age ≥65 years, being female, stage 3A CKD, and the absence of nephrology visit and comorbidities (diabetes, established cardiovascular disease, heart failure, hypertension, or chronic nephritic syndrome) were associated with undiagnosed CKD (P < 0.001). Among the diagnosed patients, the proportion receiving ≥1 prescription of guideline-recommended medications (e.g. renin-angiotensin system inhibitors) increased and their eGFR decline attenuated post-diagnosis.

**Interpretation:**

The high proportion of undiagnosed, early-stage CKD, and delayed diagnosis are concerning. The improved prescription patterns and the attenuation of eGFR decline post-diagnosis demonstrate the importance of early diagnosis and timely intervention in CKD patients.

**Funding:**

AstraZeneca China.

**Research in context**

**Evidence before this study**

Existing evidence from various countries indicates that many patients with CKD experience delayed diagnosis in clinical practice. International cohorts of REVEAL-CKD in Western Europe and North America have demonstrated high proportions of undiagnosed patients among all patients with stage 3 CKD. Although past research has reported the proportion of undiagnosed CKD (stage 3–5) at the community level in China, nationwide, up-to-date data on the undiagnosed early-stage CKD are still lacking. Additionally, limited information is available regarding patient characteristics associated with undiagnosed early-stage CKD, as well as CKD management and clinical outcomes following diagnosis among these patients.

**Added value of this study**

To the best of our knowledge, REVEAL-CKD is the first study to assess up-to-date data on the proportion of patients with, and characteristics associated with undiagnosed early-stage (stage 3) CKD at nation level in China, utilising the nationwide China Renal Data System. Our study revealed a concerningly high proportion of undiagnosed early-stage CKD within the patient population, with a low proportion of these patients receiving a diagnosis during follow-up. Age ≥65 years, being female, stage 3A CKD, and the absence of nephrology visit and comorbidities (diabetes, established cardiovascular disease, heart failure, hypertension, or chronic nephritic syndrome) were associated with undiagnosed CKD (P < 0.001). The results also demonstrated an improvement in treatment patterns and a reduction in adverse clinical outcomes post-CKD diagnosis.

**Implications of all the available evidence**

Compared with prior community-level evidence on the undiagnosed proportion of stage 3–5 CKD, the current nation-level data reveal a notably higher undiagnosed proportion of stage 3 CKD in China, highlighting poorer diagnosis of CKD at earlier stages in clinical practice. Consistent with findings from Western Europe and North America, delay (or lack thereof) in CKD diagnosis in China is similarly alarming. The observed improvement in treatment patterns and reduction in adverse clinical outcomes post-CKD-diagnosis in China also mirror those observed in the US. These findings underscore an urgent need for global efforts to enhance early CKD diagnosis and timely intervention. Additionally, understanding the characteristics associated with undiagnosed early-stage CKD in China could help inform changes in local clinical practice and healthcare policy, with the aim of increasing disease awareness and improving early diagnosis, especially among high-risk CKD populations.

## Introduction

Chronic kidney disease (CKD), defined as abnormalities of kidney structure or function present for >3 months, imposes significant burden on public health globally and in China.^1^^,^^2^ Worldwide, the 2023 update of the Global Kidney Health Atlas by the International Society of Nephrology reported a global median CKD prevalence of 9.5%.^3^ In China, a cross-sectional study estimated 82 million adults with CKD in 2018–2019, indicating a prevalence of 8.2%.^4^ Without timely intervention, CKD can progress to end-stage kidney disease (ESKD), which requires kidney replacement therapy (KRT, such as transplantation or dialysis) to sustain life, and is associated with poor prognosis and greater healthcare cost.^5^ Notably, in 2023, the global median prevalence of kidney failure on KRT was 823 per million population.^3^ The substantial burden of CKD is attributable to important risk factors including diabetes mellitus, hypertension and obesity.^2^^,^^6^ Moreover, the presence of CKD may also complicate the management of these conditions.^7^ Without adequate management for both CKD and the associated comorbidities, there is a risk of further progression, ultimately increasing the risk of all-cause and cardiovascular mortality.^7^^,^^8^

Early detection of CKD is therefore crucial for timely intervention to prevent disease progression and mitigate CKD-related adverse outcomes.^1^^,^^9^ Patients diagnosed at later disease stages have increased risks of disease progression, complication, and mortality.^10^ However, current data have suggested that many CKD patients, regardless of their disease stage, could remain undiagnosed, depriving them of the opportunities for timely intervention.^4^^,^^11^, ^12^, ^13^, ^14^, ^15^, ^16^, ^17^ In 2019, KDIGO held a controversies conference titled “Early Identification and Intervention in CKD”. The publication resulting from the conference emphasized that CKD screening and interventions should be implemented for early detection, especially among high-risk populations.^9^ Despite this, in many countries (including China), large-scale, up-to-date data regarding the proportion of undiagnosed early-stage CKD are lacking. Additionally, limited information is available regarding the clinical characteristics associated with undiagnosed early-stage CKD, as well as CKD management and clinical outcomes after diagnosis. Such data are necessary to prevent or delay disease progression and mitigate CKD-related complications and mortality.

REVEAL-CKD is a multinational, retrospective, observational study (ClinicalTrial.gov, NCT04847531) conducted in 11 countries.^18^ Using national secondary databases containing real-world patient data, this study estimated the proportion of and factors associated with undiagnosed stage 3 CKD, time to CKD diagnosis among initially undiagnosed patients, as well as CKD management and selected adverse clinical outcomes after diagnosis in the selected countries. Results gathered from the international cohorts so far have demonstrated high proportions of undiagnosed patients among all patients with stage 3 CKD, ranging from 56.0% in England to 95.5% in France.^19^^,^^20^ Here, we report the results from REVEAL-CKD in China, a retrospective cohort study of patients with stage 3 CKD from a large, nationwide database.

## Methods

### Study design

In this observational study, deidentified patient data were extracted from the China Renal Data System (CRDS). CRDS is a nationwide database currently containing data of over 8 million inpatients and outpatients who received treatment between 1 January 2000 and 31 December 2022 from 24 tertiary hospitals across China. This database was jointly established by the National Clinical Research Centre and the Chinese Centre for Disease Control and Prevention (CDC). All laboratories at the participating hospitals had passed the annual External Quality Assessment of the Chinese National Centre for Clinical Laboratories. The recorded data in CRDS included demographic characteristics, vital signs, diagnosis at admission and discharge, medication, surgical information, and laboratory measurements. To ensure data quality, a two-step quality control (QC) process was applied to both raw and standardised data. First, a random sample of raw data from each hospital was collected and reviewed to verify the accuracy and at least 95% completeness.^21^ Second, QC was conducted as feedback of the data cleaning cycles through clinical staff manually reviewing the data and submitting the QC report. The cycles repeated until the QC accuracy reached 95%. The data accuracy and completeness of CRDS had also been verified in previous studies.^21^, ^22^, ^23^, ^24^ In this study, we aimed to examine CKD diagnosis across all inpatient and outpatient departments at the hospital level. We identified patients from 20 hospitals located across five regions in China (Supplementary Table S1) between 1 January 2015 and 31 December 2020, after the exclusion of four hospitals with data from nephrology departments only. Data from the contributing hospitals were pooled and cleaned at the National Clinical Research Centre for Kidney Disease in Guangzhou, China.

**Fig. 1:**
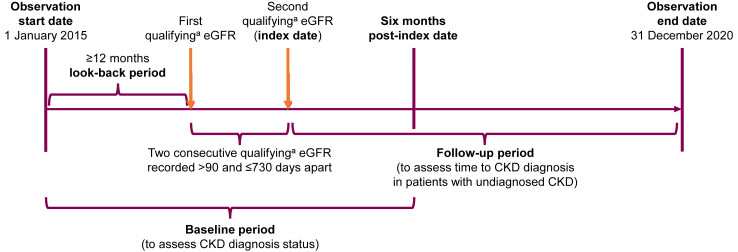
**Study timeline**. ^a^eGFR ≥30 and <60 ml/min/1.73 m^2^ using the 2009 CKD-EPI creatinine equation. CKD, chronic kidney disease; CKD-EPI, Chronic Kidney Disease Epidemiology Collaboration; eGFR, estimated glomerular filtration rate.

The study population included patients aged ≥18 years at index date, with at least two consecutive estimated glomerular filtration rate (eGFR) measurements indicating stage 3 CKD (Stage 3A or 3B; ≥30 and <60 ml/min/1.73 m^2^ using the 2009 CKD Epidemiology Collaboration [CKD-EPI] equation based on KDIGO guidelines^1^^,^^25^) recorded >90 and ≤730 days apart between 1 January 2015 and 31 December 2020. The date of the second qualifying eGFR measurement for the patient was defined as the index date, which was the earliest date that the patient's two consecutive eGFR measurements met the inclusion criteria (see Fig. 1 for study timeline). Included patients must have had at least 12 months of continuous presence in the database or registration in the data prior to the first qualifying eGFR. Patients were excluded if they had undergone solid organ transplantation, showed any evidence of advanced (stage 4 and 5) CKD based on CKD diagnostic codes, or received KRT before the index date.

The study was conducted in accordance with the Declaration of Helsinki, the International Conference on Harmonisation guidelines on Good Clinical Practice, Guidelines for Good Pharmacoepidemiology Practices, the Strengthening the Reporting of Observational Studies in Epidemiology (STROBE) guidelines, and the national regulations and guidelines governing local medical practice and ethics. The data were deidentified and protected by privacy safeguards. The study conducted in the CRDS was reviewed and approved by the Medical Ethics Committee of Nanfang Hospital of Southern Medical University (NFEC-2023-218), which waived the requirement for patient informed consent.

### Outcomes

The study primarily explored the proportion of undiagnosed stage 3 CKD among patients with stage 3 CKD and time to CKD diagnosis (defined as the time from the index date to the date of the first encounter with a CKD diagnostic code among the initially undiagnosed patients). Patients were considered undiagnosed if they lacked an ICD-10CN2016 diagnostic code for CKD (Supplementary Table S2) during the baseline period, which was defined as starting from ≥12 months prior to the first qualifying eGFR measurement and extending up to six months post index date. To assess time to CKD diagnosis, patients were followed up from the index date to the end of their follow-up due to death, loss of follow-up in CRDS, or to the study observation end date, whichever occurred first (Fig. 1).

To provide more insights into the current clinical status of undiagnosed stage 3 CKD, this study further investigated the following outcomes. Firstly, we assessed the temporal trend in the proportion of undiagnosed stage 3 CKD from 2015 to 2020 by calendar year. Secondly, we examined the baseline characteristics of patients with diagnosed and undiagnosed stage 3 CKD, including demographic and physiological/laboratory characteristics, medical history/comorbidities (by ICD-10CN2016 codes, Supplementary Table S3) and medication prescriptions (by anatomical therapeutic chemical [ATC] code, Supplementary Table S4), and analysed if there were any patient factors associated with undiagnosed stage 3 CKD. Lastly, we also explored the trend in CKD management practices (patient follow-up, rates of monitoring and meeting CKD management targets for selected quality indicators), treatment patterns (the proportion of patients receiving ≥1 medication prescription) and eGFR decline before and after a CKD diagnosis.

### Statistical analysis

The proportion of patients with undiagnosed stage 3 CKD was calculated by dividing the number of undiagnosed patients over the total number of patients included in this study. Demographic and clinical characteristics of diagnosed and undiagnosed patients were assessed at the index or the closest date within 12 months prior to the index and summarised descriptively. For a given baseline characteristic, the number and percentage of patients with missing data were reported. Time to diagnosis was estimated using the Kaplan–Meier method and reported with 95% confidence interval (CI). To evaluate the robustness of our definition for *undiagnosed* CKD, sensitivity analyses were conducted comparing a more stringent definition of CKD, as illustrated in patients with no ICD-10CN2016 codes for stage 3 or higher (sensitivity analysis 1; Supplementary Table S2), and also in patients with no ICD-10CN2016 codes based on a broader CKD definition by Winkelmayer et al. (sensitivity analysis 2; Supplementary Table S2)^26^ Time to diagnosis with each set of ICD-10CN2016 codes were estimated separately. Factors associated with undiagnosed CKD were examined using a logistic regression model. Selection of variables were based on physician's clinical experience, and univariate and multivariate analyses were conducted, with results from the latter being reported here. In multivariate analysis, covariates with a missing proportion of >20% were categorised as categorical variables. For covariates with a missing proportion of ≤20%, we assumed the missing pattern to be random and conducted multiple imputations using chained equations by generating five imputed datasets with 20 iterations each. To assess the robustness of the multivariate analysis, sensitivity analysis 3 was conducted by increasing the number of imputed datasets to 20 for covariates with a missing proportion of ≤20%. Odds ratios (ORs) with 95% CI and P values were reported for all variables in multivariate analysis.

Incidence of CKD management practices and treatment patterns within 180 days before and after CKD diagnosis among the diagnosed patients were analysed using bootstrap of 1000 samples (percentile approach). The annual eGFR decline within two years before and after CKD diagnosis was estimated among the diagnosed patients who had possessed at least two eGFR measurements recorded >6 months apart both before and after the diagnosis, and it was obtained by fitting a repeated-measures mixed-effects model. The model was adjusted for baseline eGFR, comedication use, comorbidities, and medical centres, with patients treated as random effects with an unstructured covariance structure (see Supplementary Method for a model prototype equation). Median annual eGFR decline was reported with 95% CI and compared between the two years before and after diagnosis using the Wilcoxon rank sum test. A P value <0.05 was considered statistically significant. All statistical analyses were performed using R v4.1.2.

### Role of the funding source

The study was sponsored by AstraZeneca China. The sponsor was involved in study design under the guidance of the external authors, and provided financial support for the collection, analysis and interpretation of data, and writing of the report. Data analysis and interpretation were determined by the authors, and the final content was written based on the authors’ input and direction. Authors were not precluded from accessing data in the study, and they accept responsibility to submit for publication. The decision to submit the article for publication was determined by the authors.

## Results

### Patient selection and characteristics

**Fig. 2:**
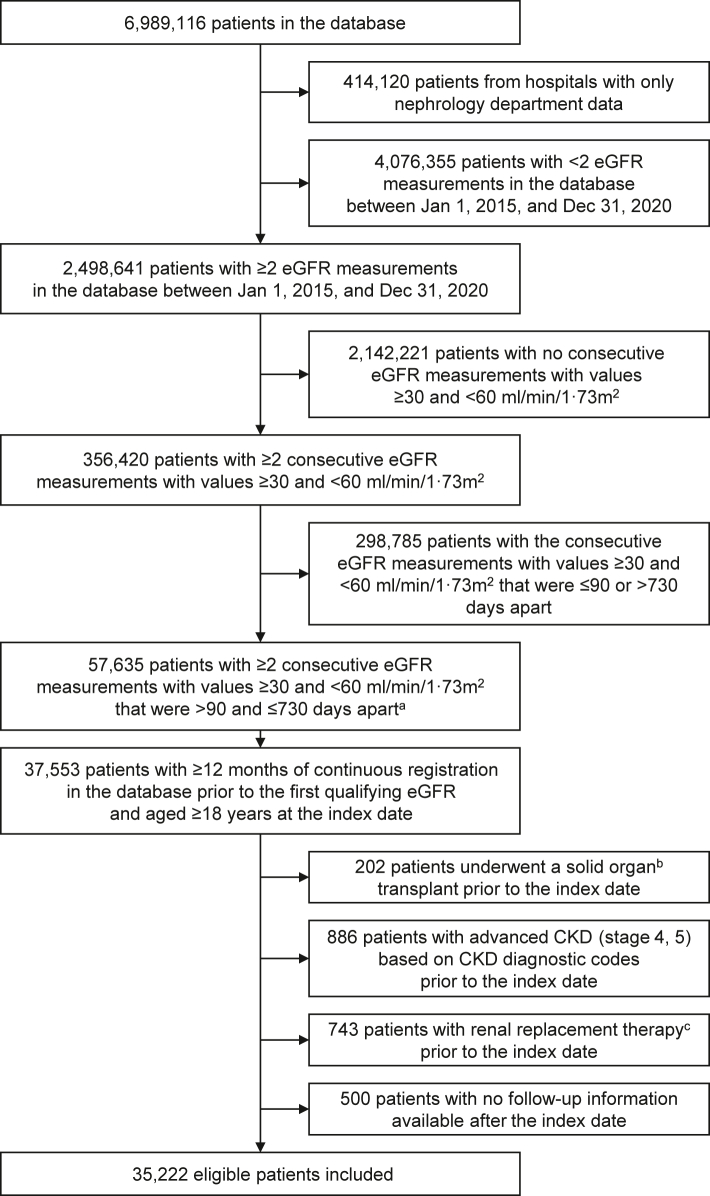
**Patient selection**. ^a^With no eGFR measurement of a value < 30 or ≥60 ml/min/1.73 m^2^ between the two qualifying eGFRs. ^b^Included kidney, liver, intestines, heart, lung, and pancreas. ^c^Included temporary and long-term dialysis. CKD, chronic kidney disease; eGFR, estimated glomerular filtration rate.

At the end of the study follow-up (31 December 2020), 35,222 patients met the inclusion criteria and were included in the analysis (Fig. 2). Patient baseline characteristics are provided in Table 1. At baseline, median (interquartile range [IQR]) age was 74.1 (64.2–81.6) years, and 19,770 (56.1%) patients were male. A total of 3899 (11.1%) patients had ≥1 nephrology visit during the baseline period. Median (IQR) eGFR was 49.0 (41.6–54.8) ml/min/1.73 m^2^, with 12,461 (35.4%) patients having eGFR of 45–<60 ml/min/1.73 m^2^ (indicating stage 3A) and 22,761 (64.6%) having eGFR of 30–<45 ml/min/1.73 m^2^ (indicating stage 3B). There were 24,235 (68.8%) patients having urine protein testing results (thereby categorised as a categorical variable), and only 2466 (7.0%) patients had available urine albumin-creatinine ratio (UACR) results. The proportions of patients with missing data for other laboratory characteristics are separately presented in Supplementary Table S5. The most common baseline comorbidities included hypertension (59.8%), established cardiovascular disease (CVD [see Table 1 for detailed definition], 39.1%), type 2 diabetes (24.7%), heart failure (23.3%), and peripheral arterial disease (21.4%). The most common baseline medications included other antihypertensive (67.5%), lipid-lowering agents (41.6%), and renin-angiotensin system inhibitor (RASi, 37.5%).

**Table 1:** Baseline characteristics of patients with diagnosed and undiagnosed stage 3 CKD.

Baseline characteristics	Overall (N = 35,222)	Undiagnosed stage 3 CKD (n = 25,214)	Diagnosed stage 3 CKD (n = 10,008)
**Patient characteristics, n (%)**			
Median age (IQR), years	74.1 (64.2–81.6)	75.3 (66.3–82.1)	70.2 (58.5–79.7)
Age group, years			
<45	1566 (4.4%)	759 (3.0%)	807 (8.1%)
45–<65	7797 (22.1%)	4857 (19.3%)	2940 (29.4%)
65–<75	9202 (26.1%)	6752 (26.8%)	2450 (24.5%)
≥75	16,657 (47.3%)	12,846 (50.9%)	3811 (38.1%)
Male	19,770 (56.1%)	13,310 (52.8%)	6460 (64.5%)
Female	15,452 (43.9%)	11,904 (47.2%)	3548 (35.5%)
Geographical region			
Southern China	23,626 (67.1%)	16,980 (67.3%)	6646 (66.4%)
Northern China	113 (0.3%)	25 (0.1%)	88 (0.9%)
Eastern China	10,242 (29.1%)	7476 (29.7%)	2766 (27.6%)
Southwestern China	787 (2.2%)	324 (1.3%)	463 (4.6%)
Northwestern China	454 (1.3%)	409 (1.6%)	45 (0.4%)
With ≥1 nephrology visit	3899 (11.1%)	1494 (5.9%)	2405 (24.0%)
Median eGFR (IQR), ml/min/1.73 m^2^	49.0 (41.6–54.8)	50.5 (43.8–55.5)	44.4 (37.1–51.7)
CKD stage			
Stage 3A (index eGFR 45–<60)	12,461 (35.4%)	7256 (28.8%)	5205 (52.0%)
Stage 3B (index eGFR 30–<45)	22,761 (64.6%)	17,958 (71.2%)	4803 (48.0%)
Urine protein^a^			
A1	16,732 (47.5%)	12,583 (49.9%)	4149 (41.5%)
A2	3212 (9.1%)	1883 (7.5%)	1329 (13.3%)
A3	2697 (7.7%)	1211 (4.8%)	1486 (14.8%)
A4	1594 (4.5%)	652 (2.6%)	942 (9.4%)
Unknown	10,987 (31.2%)	8885 (35.2%)	2102 (21.0%)
Median serum uric acid (IQR), μmol/l	425.0 (350.4–509.0)	419.0 (346.0–501.0)	439.0 (362.0–526.0)
Median total cholesterol (IQR), mmol/l	4.6 (3.7–5.5)	4.6 (3.8–5.5)	4.4 (3.6–5.4)
Median LDL cholesterol (IQR), mmol/l	2.6 (2.0–3.4)	2.7 (2.0–3.4)	2.5 (1.9–3.3)
Median serum albumin (IQR), g/l	39.7 (35.9–42.9)	40.0 (36.3–43.1)	38.9 (34.8–42.4)
**Comorbidities, n (%)**			
Established CVD^b^	13,770 (39.1%)	9485 (37.6%)	4285 (42.8%)
Heart failure	8200 (23.3%)	5239 (20.8%)	2961 (29.6%)
Hypertension	21,055 (59.8%)	13,948 (55.3%)	7107 (71.0%)
Type 1 diabetes	401 (1.1%)	165 (0.7%)	236 (2.4%)
Type 2 diabetes	8684 (24.7%)	4803 (19.0%)	3881 (38.8%)
Other diabetes^c^	1733 (4.9%)	1358 (5.4%)	375 (3.7%)
Chronic nephritic syndrome	2073 (5.9%)	786 (3.1%)	1287 (12.9%)
**Use of medications, n (%)**			
RASi	13,200 (37.5%)	8288 (32.9%)	4912 (49.1%)
Other antihypertensive medications	23,771 (67.5%)	16,300 (64.6%)	7471 (74.7%)
Lipid-lowering agents	14,652 (41.6%)	9727 (38.6%)	4925 (49.2%)
Antidiabetic agents	5774 (16.4%)	3354 (13.3%)	2420 (24.2%)

CKD, chronic kidney disease; CVD, cardiovascular disease; eGFR, estimated glomerular filtration rate; IQR, interquartile range; LDL, low-density lipoprotein; RASi, renin-angiotensin system inhibitor; UACR, urine albumin-creatinine ratio.

a Combination of UACR, 24-h urine protein excretion, 24-h urine albumin excretion, and semi-quantitative urine protein measurement and categorised into A1 (UACR <30 mg/g, 24-h urine microalbumin <30 mg/24 h, 24-h urine protein <150 mg/24 h, or urine dipstick reading of negative or trace), A2 (UACR 30–300 mg/g, 24-h urine microalbumin 30–300 mg/24 h, 24-h urine protein 150–500 mg/24 h, or urine dipstick reading of 1+), A3 (UACR >300–2200 mg/g, 24-h urine microalbumin >300–2200 mg/24 h, 24-h urine protein >500–3500 mg/24 h, or urine dipstick reading of 2+) and A4 (UACR >2200 mg/g, 24-h urine microalbumin >2200 mg/24 h, 24-h urine protein >3500 mg/24 h, or urine dipstick reading of ≥3+).

b Included history of myocardial infarction, stroke, coronary artery bypass graft, percutaneous coronary intervention, or unstable angina.

c Included any unclassified diabetes, gestational diabetes, or steroid-induced diabetes.

### Proportion of undiagnosed stage 3 CKD

**Fig. 3:**
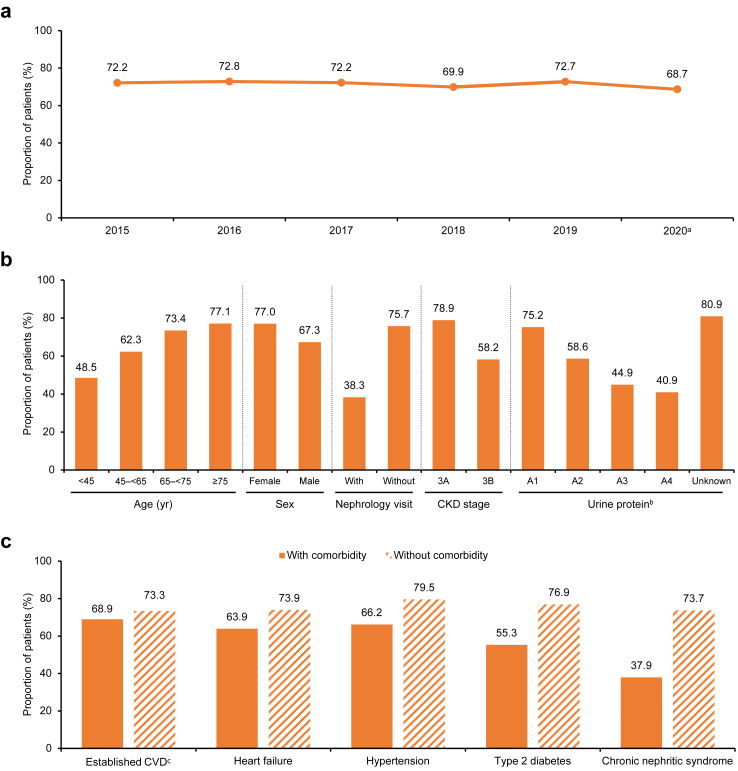
**Proportion of undiagnosed stage 3 CKD during the baseline period by (a) calendar year from 2015 to 2020; (b) key characteristic;and (c) in patients with or without comorbidity**. ^a^There were missing data for some patients after June 2020. ^b^Combination of UACR, 24-h urine protein excretion, 24-h urine albumin excretion, and semi-quantitative urine protein measurement, and categorised into A1 (UACR <30 mg/g, 24-h urine microalbumin <30 mg/24 h, 24-h urine protein <150 mg/24 h, or urine dipstick reading of negative or trace), A2 (UACR 30–300 mg/g, 24-h urine microalbumin 30–300 mg/24 h, 24-h urine protein 150–500 mg/24 h, or urine dipstick reading of 1+), A3 (UACR >300–2200 mg/g, 24-h urine microalbumin >300–2200 mg/24 h, 24-h urine protein >500–3500 mg/24 h, or urine dipstick reading of 2+), and A4 (UACR >2200 mg/g, 24-h urine microalbumin >2200 mg/24 h, 24-h urine protein >3500 mg/24 h, or urine dipstick reading of ≥3+). ^c^Included history of myocardial infarction, stroke, coronary artery bypass graft, percutaneous coronary intervention, or unstable angina. CKD, chronic kidney disease; CVD, cardiovascular disease; UACR, urine albumin-creatinine ratio.

Among the 35,222 patients included in the primary analysis, 25,214 (71.6%) patients had undiagnosed stage 3 CKD during the baseline period. The proportions of undiagnosed CKD obtained in sensitivity analyses 1 and 2 were 91.7% and 56.3%, respectively. As shown in Fig. 3a, through 2015–2020, the proportion of undiagnosed stage 3 CKD remained high (68.7%–72.8%).

### Demographic and clinical characteristics of patients with undiagnosed and diagnosed stage 3 CKD

Demographic and clinical characteristics of undiagnosed patients and diagnosed patients are provided in Table 1. In these two groups of patients, the median age was 75.3 years and 70.2 years, and the proportion of males was 52.8% and 64.5%, respectively. During the baseline period, 5.9% of the undiagnosed patients and 24.0% of the diagnosed patients had ≥1 nephrology visit. Median eGFR was 50.5 ml/min/1.73 m^2^ for the undiagnosed patients and 44.4 ml/min/1.73 m^2^ for the diagnosed patients. The proportions of patients with stage 3A and 3B CKD were 28.8% and 71.2% for undiagnosed patients, and 52.0% and 48.0% for diagnosed patients. Urine protein testing results were available in 64.8% of undiagnosed patients and 79.0% of diagnosed patients.

### Factors associated with undiagnosed stage 3 CKD

Fig. 3b and 3c illustrate the undiagnosed proportion in patients stratified by baseline characteristic and in those with or without comorbidities. As shown, the undiagnosed proportion was numerically higher in older patients compared with younger age groups (≥75 years old: 77.1%, 65–<75 years old: 73.4% vs. 45–<65 years old: 62.3%, <45 years old: 48.5%), in females than males (77.0% vs. 67.3%), in patients without nephrology visit than those with (75.7% vs. 38.3%), and in stage 3A patients compared with stage 3B patients (78.9% vs. 58.2%; Fig. 3b). For comorbidities, the undiagnosed proportion was numerically higher in patients without comorbidities at baseline than those with the comorbidity (Fig. 3c). Importantly, in the multivariate analysis (Table 2), age ≥65 years (vs. <45 years), being female (vs. male), the absence of nephrology visit (vs. the presence), stage 3A CKD (vs. stage 3B), and the absence of diabetes, established CVD, heart failure, hypertension, or chronic nephritic syndrome (vs. the presence of the abovementioned comorbidities) were significantly associated with being undiagnosed during the baseline period. Similarly in sensitivity analysis 3, these factors continued to have significant associations with being undiagnosed during the baseline period (Supplementary Table S6).

**Table 2:** Multivariate analysis of factors associated with undiagnosed CKD.

Characteristics	OR (95% CI)	P value^a^
**Patient characteristics**		
Age group (vs. <45), years		
45–<65	1.11 (0.97–1.28)	0.122
65–<75	1.81 (1.58–2.09)	<0.001
≥75	2.52 (2.19–2.91)	<0.001
Male (vs. female)	0.55 (0.51–0.58)	<0.001
With nephrology visit (vs. without)	0.30 (0.28–0.33)	<0.001
CKD stage 3B (vs. 3A)	0.37 (0.35–0.40)	<0.001
Potassium (per 1-SD increment)	0.92 (0.9–0.95)	<0.001
LDL cholesterol (per 1-SD increment)	1.03 (0.99–1.08)	0.153
Albumin (per 1-SD increment)	1.14 (1.1–1.18)	<0.001
Serum uric acid (per 1-SD increment)	0.93 (0.9–0.95)	<0.001
Haemoglobin (per 10 g/l increment)	1.17 (1.13–1.21)	<0.001
Urine protein^b^ (vs. A1)		
A2	0.72 (0.66–0.80)	<0.001
A3	0.57 (0.51–0.63)	<0.001
A4	0.55 (0.48–0.63)	<0.001
Unknown	1.24 (1.16–1.33)	<0.001
**Comorbidities (vs. no history)**		
Established CVD^c^	0.85 (0.79–0.90)	<0.001
Heart failure	0.78 (0.73–0.84)	<0.001
Hypertension	0.70 (0.66–0.75)	<0.001
Any diabetes^d^	0.51 (0.48–0.55)	<0.001
Chronic nephritic syndrome	0.65 (0.57–0.73)	<0.001
**Use of medications (vs. no history)**		
RASi	0.93 (0.87–1.00)	0.036
Other antihypertensive medications	0.84 (0.78–0.91)	<0.001
Lipid-lowering agents	0.83 (0.78–0.89)	<0.001
Antidiabetic agents	0.93 (0.85–1.01)	0.101

CI, confidence interval; CKD, chronic kidney disease; CVD, cardiovascular disease; LDL, low-density lipoprotein; OR, odds ratio; RASi, renin-angiotensin system inhibitor; SD, standard deviation.

a Logistic regression model.

b Combination of UACR, 24-h urine protein excretion, 24-h urine albumin excretion, and semi-quantitative urine protein measurement, and categorised into A1 (UACR <30 mg/g, 24-h urine microalbumin <30 mg/24 h, 24-h urine protein <150 mg/24 h, or urine dipstick reading of negative or trace), A2 (UACR 30–300 mg/g, 24-h urine microalbumin 30–300 mg/24 h, 24-h urine protein 150–500 mg/24 h, or urine dipstick reading of 1+), A3 (UACR >300–2200 mg/g, 24-h urine microalbumin >300–2200 mg/24 h, 24-h urine protein >500–3500 mg/24 h, or urine dipstick reading of 2+), and A4 (UACR >2200 mg/g, 24-h urine microalbumin >2200 mg/24 h, 24-h urine protein >3500 mg/24 h, or urine dipstick reading of ≥3+).

c Included history of myocardial infarction, stroke, coronary artery bypass graft, percutaneous coronary intervention, or unstable angina.

d Included type 1, type 2, and other subtypes of diabetes.

### Time to CKD diagnosis

Time to CKD diagnosis among all undiagnosed patients is illustrated in Supplementary Fig. S1a. Among the 25,214 patients with undiagnosed stage 3 CKD during the baseline period, only 2344 (9.3%) received a diagnosis during study follow-up, and the median (95% CI) time to CKD diagnosis among these 2344 patients was 18.1 (17.6–18.8) months for the primary analysis. Median time to CKD diagnosis obtained in sensitivity analyses 1 and 2 were 13.3 (12.9–13.7) months and 17.9 (17.3–18.8) months, respectively. The proportions of patients who received a CKD diagnosis remained lower over time in elderly patients (≥65 years old), female patients, patients with stage 3A CKD and patients without diabetes compared with their respective comparator subgroups (Supplementary Fig. S1b–e).

### CKD management quality and patient outcomes before and after a CKD diagnosis

As shown in Table 3, among the 10,008 (28.4%) patients who were diagnosed during the baseline period, the proportions of patients receiving ≥1 medication prescription within 180 days post CKD diagnosis were significantly higher compared with those prior to diagnosis. Patients receiving ≥1 prescription of RASi (including angiotensin converting enzyme [ACE] inhibitors and angiotensin II receptor blockers [ARBs]) and lipid-lowering agents had the largest numerical increases in the proportions. On the other hand, inpatient and outpatient visits and CKD management quality, including rates of monitoring and meeting CKD management targets for the selected quality indicators, were largely comparable within 180 days post and prior to CKD diagnosis (Table 3, Supplementary Table S7). Of note, the monitoring rate of glycated haemoglobin (HbA1c) and the rate of meeting CKD management targets for all parameters remained low within 180 days before and after CKD diagnosis (Supplementary Table S7).

**Table 3:** Patient follow-up and proportion of patients receiving ≥1 medication prescription within 180 days before and after CKD diagnosis.

Characteristics	Before diagnosis (n = 10,008)	After diagnosis (n = 10,008)	P value
Mean number of hospitalisations per person	1.2	0.7	<0.001
Mean number of hospitalisations in the nephrology departments per person	0.2	0.1	<0.001
Mean number of outpatient visits^a^ per person	3.2	3.5	<0.001
Proportion of patients receiving ≥1 medication prescription within 180 days (95% CI)			
ACE inhibitors	16.0% (15.2%–16.8%)	16.8% (16.0%–17.7%)	0.040
ARBs	34.4% (33.4%–35.5%)	40.0% (38.9%–41.0%)	<0.001
MRAs	23.5% (22.6%–24.4%)	25.9% (25.0%–26.8%)	<0.001
Lipid-lowering drugs	44.3% (43.2%–45.4%)	50.3% (49.2%–51.4%)	<0.001
Oral hypoglycaemic drugs	24.6% (23.7%–25.5%)	28.0% (27.1%–29.0%)	<0.001
Insulin	22.2% (21.3%–23.1%)	24.9% (24.0%–25.9%)	<0.001

ACE, angiotensin converting enzyme; ARBs, angiotensin II receptor blockers; CI, confidence interval; CKD, chronic kidney disease; MRAs, mineralocorticoid receptor antagonist.

The analysis was performed in the 10,008 patients receiving a CKD diagnostic code during the baseline period.

a Patient follow-up occurring within an interval of three days was considered as a single outpatient visit given that it usually takes 2–3 days to complete one single visit.

Among the 3619 (10.3%) diagnosed patients with at least two eGFR measurements recorded >6 months apart before and after the diagnosis, estimated annual eGFR decline within two years post diagnosis was significantly reduced compared with that prior to diagnosis (8.94 vs. 12.21 ml/min/1.73 m^2^ per year, P < 0.001; Table 4). Moreover, the proportion of patients with a rapid eGFR decline (≥4 ml/min/1.73 m^2^ per year) decreased after CKD diagnosis compared with before diagnosis, while the proportion of patients with a stable eGFR (increase or no change in eGFR) increased after CKD diagnosis than before diagnosis.

**Table 4:** Estimated annual eGFR decline within two years before and after CKD diagnosis.

Characteristics	Before diagnosis (n = 3619)	After diagnosis (n = 3619)	P value^a^
Median change in eGFR (95% CI), ml/min/1.73 m^2^ per year	−12.21 (−12.94 to −11.49)	−8.94 (−10.00 to −7.88)	<0.001
Slope group, n (%)			<0.001
Rapid decline (eGFR decline ≥4 ml/min/1.73 m^2^ per year)	2926 (80.9%)	1950 (53.9%)	
Moderate decline (eGFR decline >0 and < 4 ml/min/1.73 m^2^ per year)	364 (10.1%)	550 (15.2%)	
Stable (increase or no change in eGFR)	329 (9.1%)	1119 (30.9%)	

CI, confidence interval; CKD, chronic kidney disease; eGFR, estimated glomerular filtration rate.

The analysis was performed in a small proportion of the diagnosed patients who had at least two eGFR measurements recorded >6 months apart before and after the diagnosis.

a Repeated-measures mixed-effect model adjusted for baseline eGFR, comedication use, comorbidities, and medical centres, with patients treated as random effects with an unstructured covariance structure.

## Discussion

To our knowledge, REVEAL-CKD is the first study to show the proportion of undiagnosed stage 3 CKD and the factors associated with the undiagnosed stage 3 CKD population from an up-to-date and nationwide Chinese database, filling the data gaps for undiagnosed early-stage CKD as reflected in tertiary hospitals in China. Data from CRDS showed that a substantial proportion (71.6%) of stage 3 CKD patients were undiagnosed at baseline in the primary analysis. This high undiagnosed proportion is consistent with that observed in other REVEAL-CKD cohorts, ranging from 56.0% to 95.5%.^19^^,^^20^ This proportion was notably higher than the proportion of undiagnosed stage 3–5 CKD patients reported in a recent community-wide study (38.1%), demonstrating a poorer diagnosis proportion at an earlier disease stage.^11^ Equally concerning is the low proportion of undiagnosed patients who received a diagnosis during follow-up, which is consistently low across multiple countries (China: 9.3%; other countries 3.6%–25.9%),^19^^,^^20^ indicating serious delays in time to diagnosis for stage 3 CKD. These findings show that the lack of CKD diagnosis is similar between China and health systems in Western Europe and North America. In addition, improvement in prescription of disease modifying therapy and deceleration of eGFR decline post diagnosis were similarly observed in the China cohort and the previous US cohort,^27^ further highlighting the importance of global efforts to improve recognition of CKD.

A concerningly high level of undiagnosed stage 3 CKD (>50%) was consistently observed in both the primary analysis and sensitivity analyses 1 and 2, highlighting the seriousness of the undiagnosis issue, despite some numerical differences in the undiagnosed proportion being noted between the primary analysis and the sensitivity analyses. These differences had been expected due to the different diagnostic criteria applied in each analysis. In the primary analysis, patients meeting CKD diagnostic criteria based on the ICD-10CN2016 codes, including the sub-classifications, were considered diagnosed. In sensitivity analysis 1, more stringent diagnostic criteria were used, which required a clear staging by physicians to confirm diagnosis, thereby greatly increasing the undiagnosed proportion (91.7%) compared with the primary analysis. In sensitivity analysis 2, a broader CKD definition by Winkelmayer et al. was used,^26^ which led to a lower undiagnosed proportion (56.3%) than the primary analysis.

High proportions of undiagnosed stage 3 CKD patients may lead to missed opportunities for early intervention and mitigation of disease progression, resulting in increased risks of disease complication and mortality.^1^^,^^9^ A study in Denmark showed that the risk of heart failure and even mortality increased gradually from stage 1–2 CKD to stage 3 and more advanced stages.^28^ Another Chinese study also demonstrated that the risks of ESKD, cardiovascular events and associated deaths, and all-cause mortality increased with decreasing eGFR among patients with stage 1–4 CKD.^27^ Additionally, CKD disease progression is associated with increased healthcare resource use and costs.^29^ Therefore, it is imperative to formulate strategies to improve early CKD diagnosis for the implementation of timely intervention. The considerable population differences in the undiagnosed proportions observed across the different REVEAL-CKD cohorts are likely attributed to variations in local healthcare systems and CKD management patterns. Systematic studies are needed to examine the country-level differences in clinical practice and to identify effective management strategies to improve CKD diagnosis.

The processes of CKD screening, diagnosis and management may be poorly defined, especially in primary care settings, resulting in low awareness among physicians and missed opportunities for early detection. A survey conducted in 12 community health centres in Beijing revealed a low awareness rate (17%–25%) of CKD staging, diagnosis, prevention, and treatment among general physicians.^30^ This may be due to a lack of a hierarchical, institutionalised framework for CKD management. Disease education and clinical practice guidance are needed, especially for general physicians, to enhance CKD diagnosis and management. Furthermore, cooperation between primary care and central hospitals may help empower general physicians in primary care. In 2022, the Chinese National Health Commission released a technical guidance for hierarchical diagnosis and treatment for CKD in county areas. Besides providing guidance on CKD screening, diagnosis and management, it also outlines the roles of healthcare providers at different levels (village, county, city, etc.) and how they should cooperate to improve diagnosis and linkage to care, establishing a robust framework for hierarchical management of CKD.^31^ Such guidance can help facilitate knowledge exchange and provide critical tools to empower primary care providers and enhance the integrated care of CKD.

Another important observation is the low availability of urine protein results (68.8%), particularly UACR (7.0%), which is similar to that observed in the other international cohorts (ranging from 1.8% in the US to 5.5% in Japan).^19^ Insufficient urine protein testing has existed for years in clinical practice, corroborated by a 21.0% UACR testing rate for CKD-at-risk patients in the US and a 19.4% testing rate for albuminuria (normally using UACR testing) among high-risk CKD population in China.^11^^,^^32^ This low testing rate could be due to insufficient physician attention to early screening and detection of CKD, potentially leading to a low test prescription rate. Additionally, it may be attributed to low testing accessibility. Xu et al. reported that UACR testing was only available in <20% of the 79 community health centres in Xicheng District, Beijing.^11^ Concerted effort is warranted to facilitate education among physicians on the significance of quantitative urine protein testing, and to improve testing accessibility for earlier CKD detection, especially for high-risk populations.

Interestingly, this study observed a numerically much lower undiagnosed proportion in patients with chronic nephritic syndrome than those without (37.9% vs. 73.7%, Fig. 3c). Patients with nephritis typically have their initial consultation in the nephrology department, potentially contributing to a lower likelihood of missed CKD diagnoses. Literature has historically indicated that glomerulonephritis was the predominant cause of CKD in developing countries, while diabetes and hypertension were more common causes in developed countries.^33^, ^34^, ^35^ This may also explain why the proportion of undiagnosed stage 3 CKD in the Chinese cohort (71.6%) lies at the lower middle range observed across the international cohorts (56.0%–95.5%) of REVEAL-CKD.^19^^,^^20^ However, it is worth noting that the etiology of CKD in China has been changing in recent years alongside economic development and socioenvironmental changes. There is an increasing trend of CKD caused by diabetes, while glomerulonephritis-related CKD is decreasing.^34^ If this trend persists, there might be fewer patients visiting the nephrology department as their first point of care, potentially leading to an increase in undiagnosed early-stage CKD. Therefore, similar to that emphasized by international consensus documents,^36^^,^^37^ it is crucial to raise awareness of CKD and include relevant testing in healthcare units other than nephrology departments in China, especially in the endocrinology department to improve early diagnosis and intervention of diabetes-related CKD, as well as that associated with other etiologies.

Compared with younger age groups, elderly patients had a higher proportion of undiagnosed stage 3 CKD and experienced longer time to diagnosis (Fig. 3b, Supplementary Fig. S1b), likely due to a much lower disease awareness (45–59 years: 27.3%, 60–79 years: 8.7%).^38^ Notably, the impact of delayed diagnosis, or lack thereof, among elderly may become more pronounced with an aging population in China, given that the proportion of elderly (≥65 years old), being 9.7% in 2013 and 13.5% in 2020,^39^ is projected to further increase to 18.2% by 2030 and 30.1% by 2050.^40^ Taken together, it is imperative to raise awareness of CKD and increase CKD screening rate among elderly patients, possibly through targeted education and decentralisation of CKD-related services to primary healthcare to improve accessibility for the elderly, especially for those with limited mobility.^41^

The proportion of patients receiving ≥1 prescription of guideline-recommended, risk-modifying therapies for CKD management generally improved after a CKD diagnosis, especially for RASi (Table 3), reflecting an increase in treatment willingness post diagnosis. RASi has been used for blood pressure control and kidney function preservation in CKD patients.^42^^,^^43^ Its increased prescription could delay eGFR decline and reduce the risk of adverse clinical outcomes. Such interpretation was preliminarily reflected by the significantly attenuated eGFR decline post diagnosis (Table 4), though this was only observed in a small proportion of patients with a CKD diagnosis and further validation in a larger sample would be warranted. However, the rates of meeting CKD management targets for laboratory parameters were generally low (Supplementary Table S7). This is not particularly surprising, as firstly, the rate of meeting management targets for chronic diseases such as diabetes mellitus and hypertension is generally below 50%.^34^ Secondly, it may take longer than 180 days (6 months) for such improvements to be evident. Such results indicate a need for more effective management of CKD and other related chronic conditions.

This study presents some limitations listed here. Firstly, only tertiary hospitals were included in the CRDS, which limits the representativeness of the study for community populations and primary care settings. Nevertheless, the CRDS contains nationwide patient data, enabling robust analyses with a substantial sample size that covers major administrative regions, diverse patient demographics and various comorbidities across China. Thus, our study would still provide credible insights into the diagnostic status of stage 3 CKD within China. Secondly, although the aim of this study was to assess stage 3 CKD patients, including only this stage may limit the generalisability of these results to other early-stage CKD patient groups (i.e. stage 1 and 2), where diagnosis largely relies on albuminuria screening and therefore is often missed due to inadequate testing for albuminuria.^9^^,^^44^ Thirdly, alternative indicators related to disease diagnosis, such as urine protein, were not captured in sufficient numbers and could not be used for CKD identification. Fourthly, in theory, a patient could transfer to another region for care, resulting in double counting in the CRDS. However, the probability of such double counting should be quite low as few patients would transfer across province to seek medical treatment for chronic diseases. Lastly, missing data for some clinical characteristics were reported in this study. Although imputations were conducted to address missing data for covariates with a missing proportion of ≤20% in the multivariate analysis, caution may be needed when interpreting results for such characteristics due to potential biases introduced by missing data.

In conclusion, using this large, representative patient sample from the CRDS database, this study demonstrates a concerningly high proportion of undiagnosed early-stage CKD in the general patient population in hospitals, despite indications of a diseased state based on eGFR measurements. The observed improvement in treatment patterns and the reduction in adverse clinical outcomes following CKD diagnosis signify the crucial role of diagnosis in allowing the initiation of appropriate interventions and managing the condition effectively. Taken together, these results highlight the critical need to enhance disease awareness and improve early diagnosis, ensuring that more patients receive timely interventions to prevent further disease progression and mortality.

## Contributors

SN and FFH conceptualised the study. SZhou and SC acquired the data. SZhou, CC, SC, and SZhang performed data analysis. CC, SC, MJ, SZhang, LS, QG, and NT performed data interpretation. FFH supervised the study. SN, CC, and MJ drafted the manuscript, while SZhou, SC, SZhang, LS, QG, NT, and FFH reviewed and edited the manuscript. SN, SZhou, CC, SC, MJ, SZhang, and FFH have accessed and verified the data. All authors had full access to all the data in the study, and had final responsibility for the decision to submit the manuscript for publication.

## Data sharing statement

The data presented in this study are available on request from the corresponding author.

## Declaration of interests

SN and FFH received honoraria from George Clinical. CC is an employee of AstraZeneca and has shareholder interest in the company. MJ and SZhang are employees of AstraZeneca. NT received grants from CIHR, NIH, Kidney Foundation of Canada, Bayer, AstraZeneca, Boehringer Ingelheim, Janssen Pharmaceuticals, Research Manitoba, Otsuka Pharmaceutical Co, Ltd, Tricida and Eli Lilly and Company; consulting fees from AstraZeneca, Bayer, Boehringer Ingelheim, GSK, Janssen Pharmaceuticals, Otsuka Pharmaceutical Co, Ltd, Prokidney, Roche and Tricida Inc; honoraria from AstraZeneca, Boehringer Ingelheim, Eli Lilly and Company, Janssen Pharmaceuticals, Otsuka Pharmaceutical and Tricida Inc. NT holds a pending patent on a microfluidic device for point of care detection of urine albumin, serves a leadership or fiduciary role in advisory boards for AstraZeneca, Janssen Pharmaceuticals, Boehringer Ingelheim/Eli Lilly and Company, Otsuka Pharmaceutical Co, Ltd and National Kidney Foundation, and holds stock or stock options from ClinPredict, Klinrisk, Quanta, Marizyme, Mesentech, Rénibus Therapeutics, pulseData and Tricida. All other authors have no conflict of interest to declare.

## Appendix A Supplementary data

Statement of Role of Medical Writer

Protocol Desensitization
