# Supplementary material for: Characteristics of patients with undiagnosed stage 3 chronic kidney disease: results from an observational study (REVEAL-CKD) in China
Source: Lancet Reg Health West Pac. 2025 Jan 4;54:101275. doi: 10.1016/j.lanwpc.2024.101275 (PMC11758424; doi:10.1016/j.lanwpc.2024.101275)
Supplement: Protocol Desensitization [file mmc2.pdf]

Observational Study Protocol

Study Code	D169AR00003
Version	1.0 China
Date	24 April 2023

REVEAL-CKD: Prevalence and consequences of undiagnosed chronic kidney disease

A multinational observational study to determine the prevalence and consequences of undiagnosed chronic kidney disease

Sponsor: AstraZeneca

Global Author: [redacted]

Local Author: [redacted]

TABLE OF CONTENTS PAGE

TABLE OF CONTENTS 2
LIST OF ABBREVIATIONS AND DEFINITION OF TERMS 4
PROTOCOL SYNOPSIS 6
1. BACKGROUND AND RATIONALE 9
1.1 Background 9
1.2 Rationale 9
2. OBJECTIVES AND HYPOTHESES 10
3. METHODOLOGY 11
3.1 Study Design – General Aspects 11
3.2 Data Source 11
3.3 Study Population 13
3.4 Study Selection Criteria 13
3.5 Participant Follow-up 14
4. VARIABLES AND EPIDEMIOLOGICAL MEASUREMENTS 14
4.1 Exposures 14
4.2 Outcomes 14
4.3 Other Variables and Covariates 16
4.3.1 Baseline Patient Characteristics and Comorbidities 17
4.4 Study Subgroups 19
5. STATISTICAL ANALYSIS PLAN 20
5.1 Statistical Methods – General Aspects 20
5.2 Statistical Analyses 20
5.3 Bias 22
5.3.1 Methods to Minimize Bias 22
5.3.2 Adjustment for Multiple Comparisons 23
5.3.3 Study Limitations 23
5.4 Interim Analyses (Optional) 23
5.5 Sample Size and Power Calculations 24
6. STUDY CONDUCT AND REGULATORY DETAILS 24
6.1 Study Conduct 24
6.1.1 Procedures 24
6.1.2 Quality Control 24

6.2 Protection of Human Subjects....24
6.2.1 Subject Informed Consent (Primary Data Collection Only)....25
6.2.2 Confidentiality of Study/Subject Data (Primary Data Collection Only)....25
6.3 Collection and Reporting of Adverse Events/Adverse Drug Reactions....25
7. LIST OF REFERENCES....26
8. APPENDICES....27
9. SIGNATURE....29

LIST OF ABBREVIATIONS AND DEFINITION OF TERMS

Abbreviation or special term	Explanation
ACE	Angiotensin-converting enzyme
ARB	Angiotensin II receptor blockers
ARNI	Angiotensin receptor-neprilysin inhibitor
ASCVD	Atherosclerotic cardiovascular disease
BMI	Body mass index
CABG	Coronary artery bypass graft
CI	Confidence interval
CKD	Chronic kidney disease
CRDS	Chinese Renal Disease Data System
CRT	Cardiac resynchronization therapy
DPP-4i	Dipeptidyl peptidase 4 inhibitors
eGFR	Estimated glomerular filtration rate
EMR	Electronic medical record
EU	European Union
GCP	Good Clinical Practices
GLP1-RA	Glucagon-like peptide-1 receptor agonist
GPP	Guidelines for Good Pharmacoepidemiology Practices
ICD	International Classification of Diseases
ICD	Implantable cardioverter-defibrillator
ICH	International Conference on Harmonisation
IEC	Independent Ethics Committee
IHD	Instant Health Data
IQR	Interquartile range

Abbreviation or special term	Explanation
IRB	Institutional Review Board
LDL	Low-density lipoprotein
MI	Myocardial infarction
MRA	Aldosterone receptor agonists
MDRD	Modification of Diet in Renal Disease
PCI	Percutaneous coronary intervention
SD	Standard deviation
T1D	Type 1 diabetes
T2D	Type 2 diabetes
TRH	Treatment-resistant hypertension
UA	Unstable angina
UACR	Urine albumin creatinine ratio
MAPD	Medicare Advantage and Prescription Drug
US	United States
UK	United Kingdom

PROTOCOL SYNOPSIS

REVEAL-CKD: A multinational observational study to determine the prevalence and consequences of undiagnosed chronic kidney disease

Background/Rationale: The diagnosis of chronic kidney disease (CKD) occurs predominantly during advanced disease (stages 4, 5) when there are few opportunities to delay further progression and avoid complications. Detection of CKD to optimize patient outcomes requires active screening and monitoring in the early stages of CKD (3A or 3B), especially in at-risk asymptomatic patients, such as those with existing type 2 diabetes (T2D) and treatment-resistant hypertension (TRH). Early disease interventions can slow CKD progression that may ultimately translate into significant cost savings and, more importantly, reduce the risk of complications and the number of patients developing end-stage kidney failure. Low diagnosis rates of CKD at early stages have been reported in the United States (US)[1], but this has not been broadly studied in other countries. Moreover, the demographic and clinical predictors of undiagnosed CKD remain to be determined.

Objectives:

Primary Objectives

1) Estimate the point prevalence of undiagnosed stage 3 CKD (proportion of patients with eGFR measurements indicating stage 3 CKD with no corresponding CKD diagnostic code either before or up to six months after the second abnormal eGFR value)
2) Describe time to CKD diagnosis in patients with no prior CKD diagnosis code at index date (time of second qualifying eGFR), overall and by patient characteristics

Secondary Objectives

1) Assess trends in the prevalence (point prevalence) of undiagnosed CKD by calendar year
2) Describe baseline characteristics (at index date) among those with undiagnosed versus diagnosed CKD
3) Assess CKD management and monitoring practices (post index date) in patients with diagnosed versus undiagnosed CKD

Methods:

Study design: The study will be a multinational, non-interventional, observational study. A series of cohort studies will be conducted to assess the prevalence of undiagnosed stage 3 CKD using eGFR laboratory measurements and diagnostic codes. The study will also assess the current state of CKD management . Relevant data sources (e.g. claims, electronic medical records [EMR]) will be identified in the countries of interest.

Study Population: All patients ≥18 years old with two consecutive eGFR laboratory measurements indicating stage 3 CKD (eGFR 30–59 mL/min/1.73m^2 using CKD-EPI [preferred] or MDRD equation) recorded at least three months apart during the observation period will be included in the study and constitute the denominator for the calculation of the prevalence of undiagnosed stage 3 CKD. The date of the second qualifying eGFR will be considered as the index date. A subset of the study population will be identified with no encounters with a diagnosis code for CKD any time prior to the study index date (for primary objective 2). The observation period will vary based on the period of data coverage, which will be unique to each data source.

Case definition for undiagnosed stage 3 CKD

Patients with undiagnosed stage 3A-3B CKD will be identified if they have no healthcare encounter with a diagnosis code for CKD at any time during the ≥12-month lookback period before the first eGFR measurement and up to 6 months post-index date. Those with a documented diagnosis of CKD during this time period will be considered as having diagnosed CKD.

CKD management and monitoring

To assess the current state of CKD management and monitoring practices by CKD diagnosis status, information on quality indicators[2] will be extracted in the following seven domains starting 6 months post index date:

1. Detection and recognition of CKD (e.g. UACR testing within 6 months)
2. Monitoring of kidney function and complications (e.g. serum creatinine measurement within 18 months)
3. Use of recommended medications (e.g. ACEIs, ARBs or SGLT2i)
4. Kidney function monitoring after initiation of ACEIs ARBs
5. Kidney function monitoring after initiation of SGLT2i
6. Management of blood pressure
7. Monitoring of glycaemic control

Statistical Analysis: The prevalence of undiagnosed stage 3 CKD will be calculated. The prevalence numerator will include all patients who fulfill the undiagnosed stage 3 CKD case

definition at any time during the observation period. The denominator will include all individuals included in the study (cohort 1).

Prevalence will be estimated by dividing the numerator by the denominator, and 95% confidence intervals (CI) around prevalence will be estimated using a binomial distribution. Prevalence will be assessed during the observation period and by each calendar year of the index date. Prevalence will also be calculated among subgroups of patients based on age, sex, and the presence of key baseline comorbidities. For those with no CKD diagnosis code on or before the index date (cohort 2), time to CKD diagnosis will be assessed using KM curve.

Baseline characteristic (at index date) will be described for undiagnosed versus diagnosed CKD. The proportion of patients achieving each of the specified quality indicators will be described by CKD diagnosis status, overall and by comorbidities.

Sample Size Estimation: If the prevalence rate of undiagnosed CKD is between 60% and 80%, then a sample of 1,000 patients will be sufficient to estimate the prevalence rate with a margin of error of +/-3% in a 95% CI per institution/country (6% width of 95% CI).

According to the feasibility assessment of REVEAL-CKD in China, the number of patients who meet the inclusion and exclusion criteria in the Chinese Renal Disease Data System (CRDS) database is about 27,000 and the number of patients who met the inclusion and exclusion criteria of sensitivity analysis in the CRDS database is about 46,000[3].

1. BACKGROUND AND RATIONALE

1.1 Background

Chronic kidney disease (CKD) describes the gradual loss of kidney function over time. It is typically defined using indicators of kidney function, such as the estimated glomerular filtration rate (eGFR). Usually, patients are diagnosed with CKD if reductions in kidney function are present for more than 3 months.[4] Most people who have a mild-to-moderate (stage 3A, eGFR 45–59 ml/min/1.73m^2) or moderate-to-severe reduction in kidney function (stage 3B, eGFR 30–44 ml/min/1.73m^2) are asymptomatic and not aware of even having CKD.[5] According to a study from the United Kingdom (UK), CKD stage 3 is estimated to contain the largest proportion of cases with over 90%.[6]

The global incidence of stage 3–5 CKD in 2016 was 21 million, representing an 88.8% increase in incidence in the last 27 years (11 million in 1990). The CKD incidence rate per 100,000 persons increased from 214.6 in 1990 to 288.5 in 2016[7], whereas the age-standardized incidence rate increased from 299.1 to 310.1 per 100,000 population during that same period. While the increasing incidence is partly related to aging populations, it is also associated with an increased prevalence of key risk factors, including diabetes and hypertension.[8]

Treatment for CKD focuses on slowing the progression of kidney damage, usually by a combination of measures to control the underlying cause or to reduce complications.[9] For example, medications such as angiotensin-converting enzyme (ACE) inhibitors or angiotensin II receptor blockers (ARB) are used to treat worsening blood pressure and preserve kidney function. Statins are also recommended to lower cholesterol levels, as individuals with CKD often have high levels of low-density lipoprotein (LDL) cholesterol, which is a risk factor for heart disease. Early treatment is important because, even at the earliest stages (stage 3A and 3B), CKD is regarded as an independent risk factor for all-cause death and cardiovascular events (Go et al., 2008).[10] In addition, CKD can progress to end-stage kidney failure, which is fatal without artificial filtering (dialysis) or a kidney transplant.[9] Even with dialysis, mortality rates remain above 20 percent per year (Go et al., 2008) and quality of life is significantly reduced.

1.2 Rationale

The diagnosis of CKD occurs predominantly during advanced disease (stages 4, 5), when there are few opportunities to delay further progression and avoid complications. Detection of CKD to optimize patient outcomes requires active screening and monitoring in the early stages of CKD (3A or 3B), especially in at-risk asymptomatic patients, such as those with existing type 2 diabetes (T2D) and treatment-resistant hypertension (TRH). Any effective action to slow CKD progression depends on early disease recognition that ultimately will translate into significant cost savings and, more importantly, reduce the risk of complications and the number of patients developing end-stage kidney disease. Low diagnosis rates of CKD at early stages have been reported in the US [1], but this has not been broadly studied in other

countries. Moreover, the demographic and clinical predictors of undiagnosed CKD remain to be determined.

2. OBJECTIVES AND HYPOTHESES

The overall aim of this study is to describe the prevalence and predictors of undiagnosed stage 3 CKD in priority countries.

Table 1. Study objectives

**Primary objectives:**	**Outcome measure**	**Hypothesis tested (if relevant)**
1. Describe the point prevalence of undiagnosed stage 3 CKD,	Point prevalence of undiagnosed CKD stage 3<br><br>Prevalence of undiagnosed CKD defined as proportion of: (1) Patients with eGFR 30–59 (inclusive) and no diagnosis code for CKD divided by (2) all patients with eGFR 30–59<br><br>It will be assumed that patients with at least one diagnostic code for CKD have diagnosed CKD	Not applicable
2. Describe time to CKD diagnosis in patients with no prior CKD diagnosis code at index date (time of second qualifying eGFR)	Median time to CKD diagnosis from index date. KM curve, overall and by patient characteristics	
**Secondary objectives:**	**Outcome measure**	**Hypothesis tested (if relevant)**
1. Assess trends in the prevalence of undiagnosed CKD by calendar year of the index date	Prevalence of undiagnosed CKD by calendar year	Not applicable
2. Describe baseline characteristics at index date among those with	Baseline patient characteristics and comorbidities by CKD diagnosis status	

diagnosed versus undiagnosed CKD		
3. Assess CKD management and monitoring practices (post index date) in patients with diagnosed versus undiagnosed CKD	Proportion of patients achieving each quality indicator within the following domains six months post-index date:<br>1. Detection and recognition of CKD (e.g. UACR testing within 6 months)<br>2. Monitoring of kidney function and complications (e.g. serum creatinine measurement within 18 months)<br>3. Use of recommended medications (e.g. ACEIs or ARBs)<br>4. Kidney function monitoring after initiation of ACEIs ARBs SGLT2i<br>5. Management of blood pressure<br>6. Monitoring of glycaemic control	

CKD, chronic kidney disease; eGFR, estimated glomerular filtration rate; T2D, type 2 diabetes

3. METHODOLOGY

3.1 Study Design – General Aspects

This study is a multinational, non-interventional, retrospective observational study. The study does not attempt to test any specific *a priori* hypothesis; it is descriptive only and will collect data under conditions of routine medical care. Relevant secondary databases will be identified, and a series of cohort studies will be conducted to assess the prevalence of undiagnosed CKD. The study will also assess the current state of CKD management associated with undiagnosed CKD. Undiagnosed cases are those with eGFR measurements indicating stage 3 CKD with no corresponding CKD diagnostic code (e.g. International Classification of Diseases (ICD) 9 or 10).

3.2 Data Source

Relevant secondary databases containing routinely collected data, including electronic medical records (EMR) and healthcare claims, will be identified in priority countries that meet the following minimum criteria:

1) Data availability spanning at least two calendar years
2) Recorded laboratory result values on eGFR and availability of diagnosis codes or other variables to determine physician-diagnosed CKD

3) Claims data or records of primary care and/or outpatient consultations to capture CKD diagnosis using relevant coding (e.g. ICD 9/10)

Initially, we anticipate utilizing retrospective data listed in
Table 2. Initial countries to be considered for REVEAL-CKD include the US, Spain, France, Italy, Germany, Brazil, Australia, Canada, UK, China, and Japan, however, this may be subject to change. REVEAL-CKD will be conducted in three stages (

Table 2).

Table 2. Potential data sources*

	**Country**	**Database**	**In-house availability**
Stage 1	US	IBM LCED	Yes
	US	TriNetx	Yes
	US	Humana	No
	Japan	Japan RWD	Yes
	France	The Health Improvement Network	Yes
	Germany	Academic database/Disease analyser	Yes
Stage 2	Spain	BIGPAC	No
	Italy	Health Search Database/SHENZHOU	No
Stage 3	China, Brazil, Australia, Canada and UK		

UK, United Kingdom; US, United States; LCED, Linked Claims and EMR Data. *Data sources are subject to change; No in-house data available for China.

Data sources

Data sources are listed in Table 2 above and describe whether data source is available through an existing AZ license. The database selected for China is described below.

CRDS Database (China)

In December 2018, the National Clinical Research Center for Kidney Disease, the Center for Chronic Non-communicable Disease Prevention and Chinese Center for Disease Control and Prevention jointly established the Chinese Renal Disease Data System (CRDS). By the end of

November 2022, the CRDS database contained 24 Grade 3A hospitals, including 16,953,679 patients with data collected from 1994 to 2022, and consisting mostly of hospital-wide data. Data governance has been completed in 19 hospitals with 15,245,055 patients.[3] The data of rest 5 hospitals has been governed in the CRDS database after 2022 and given the characteristics and of the CRDS database, we will exclude hospitals with data only from nephrology departments; with 20 remaining hospitals that will be included. Observation start date will be January 1 2015 and observation end date will be 31 December 2020 according to quality and situation of CRDS database.

3.3 Study Population

Patients ≥18 years old with two consecutive eGFR measurements indicating stage 3 CKD (≥30 and <60 mL/min/1.73m^2 using CKD-EPI (preferred) or MDRD equation) recorded more than 90 days apart (max. 730 days), meeting the inclusion criteria will be included in the study and constitute the denominator for the calculation of the prevalence of undiagnosed stage 3 CKD. A subset of the study population will be identified with no encounters with a diagnosis code for CKD any time prior to the study index date (for primary objective 2). The observation period will vary based on the period of data coverage, which may be unique to each data source. In CRDS database observation start date will be January 1, 2015, and observation end date will be 31 December 2020.

3.4 Study Selection Criteria

The study selection criteria are outlined below:

Inclusion criteria

1. At least two consecutive eGFR laboratory tests with values ≥30 and <60 mL/min/1.73 m^2 (Stage 3A or 3B) that are >90 and ≤730 days apart. The index date is the date of the second eGFR measure meeting the criteria for stage 3 CKD

2. At least 12 months of continuous presence in the database or registration in the data prior to the first qualifying eGFR (for data sources with information on enrollment)

3. Age ≥18 years at index date

Exclusion criteria

Patients will be excluded if they meet the following criteria:

1. Solid organ transplant before the study index date
2. Any evidence of advanced CKD (stage 4, 5) based on CKD diagnostic codes, or renal replacement therapy before the index date

3.5 Participant Follow-up

CKD diagnosis status will be assessed using data from the ≥12-month baseline period until six months post-index for the calculation of undiagnosed CKD prevalence. To assess time to CKD diagnosis (primary objective 2), patients will be followed from the index date to the earliest of their follow-up end date (due to death or transfer out of database) or end of data coverage period

To assess CKD management and monitoring quality indicators (secondary objective 3), patients will be followed starting from the six months post index date until the follow-up end date (due to death or transfer out of the database) or end of data coverage, whichever comes first.

4. VARIABLES AND EPIDEMIOLOGICAL MEASUREMENTS

4.1 Exposures

Not applicable; there are no drug exposures in this study.

4.2 Outcomes

Case Definition – Undiagnosed Stage 3 CKD

Patients with undiagnosed stage 3A-3B CKD will be identified if they have no healthcare encounter with a diagnosis code for CKD at any time during the ≥12-month lookback period before the first eGFR measurement and up to 6 months post-index date (baseline period). Those with a documented diagnosis of CKD during this time period will be considered as having diagnosed CKD. It will be assumed that patients with at least one diagnostic code for CKD during the above specified time window have diagnosed CKD. **错误!未找到引用源。** below shows the study schematic.

Figure 1. Study suggested schematic(s) below

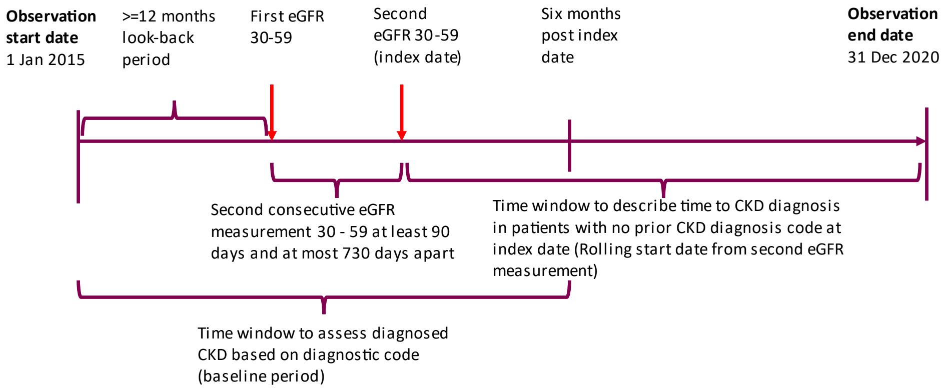

The total number of the hospitals will be included is 20 (excluding the 4 hospitals with only nephrology data)

CKD, chronic kidney disease; eGFR, estimated glomerular filtration rate

CKD management and monitoring

To assess the current state of CKD management and monitoring practices by CKD diagnosis status, information on quality indicators will be extracted in the following seven domains[2] starting six-months post index date.

1. Detection and recognition of CKD

- Patients receiving a UACR test within first 6 months of follow-up

2. Monitoring of kidney function and complications

Patients receiving the following tests within first 18 months of follow-up

- Serum Cr test (outpatient)
- Patients receiving a UACR test (outpatient)
- Serum calcium
- Phosphate
- Albumin
- Bicarbonate
- Potassium
- Hemoglobin
- Albuminuria

3. Use of recommended medications

- Statin prescription within one year
- ACEis or ARBs within one year
- SGLT2 in patients with diabetes
- SGLT2 in patients without diabetes

4. Kidney function monitoring after initiation of ACEIs or ARBs

- Patients who receive an outpatient serum creatinine measurement 7-30 days after initial ACEi or ARB prescription

5. Kidney function monitoring after initiation of SGLT2i

- Patients who receive an outpatient serum creatinine measurement 7-30 days after initial SGLT2i prescription

6. Management of BP

- Patients receiving BP measurement any time during follow-up
- At least one BP measurement within 6 months
- In patients with least one BP measurement within 6 months (starting 6 months post index), patients with at least one BP measurement ≤140/90 mm Hg in the first or second year of follow-up
- In patients with least one BP measurement within 6 months (starting 6 months post index), patients with at least one systolic BP measurement <120 mm Hg in the first or second year of follow-up

7. Monitoring of glycaemic control

- Patients with a history of diabetes prior to the index date receiving an HbA1c test within the first and second year of follow-up

4.3 Other Variables and Covariates

The following baseline variables will be examined. Unless otherwise specified, medical history/comorbidity will be assessed using all available data prior to the index date. Baseline physiological and laboratory values will be extracted at the point of index date or using the most recent values during the 12 months before the index date. Similarly, medication use will be ascertained on or within 12 months before the index date.

4.3.1 Baseline Patient Characteristics and Comorbidities

The following baseline characteristics will be assessed:

- *Demographic variables*
- o Age in years at index date, continuous and categorical (<45, 45-<65, 65-<75, >=75 years)
- o Sex, categorical (female, male, not available)
- o Ethnicity (or education if available) categorical
- o Family history of CKD
- o Smoking status

- *Physiological/laboratory values*
- o Height (cm), continuous
- o Weight (kg), continuous
- o Body mass index (BMI), continuous, calculated as kg/m^2 where *kg* is weight and *m* is height
- o Hemoglobin (Hb), g/dL
  - o Hb >12
  - o Hb 10-12
  - o Hb 8-10
  - o Hb <8
- o Hematocrit
  - o <40%
- o eGFR ($mL/min/1.73m^2$) using CKD-EPI or MDRD equation, continuous
- o Potassium, highest and closest to index date within 1 year prior to index, mmol/l
  - o 5.5–5.9 (mild hyperkalemia)
  - o 6.0–6.4 (moderate hyperkalemia)
  - o >6.5 (severe hyperkalemia)
- o Urine albumin creatinine ratio (UACR, mg/g), continuous, calculated as urine albumin (mg/dL) / urine creatinine (g/dL)
- o Serum uric acid levels, continuous
- o Total cholesterol, continuous
- o Low density lipoprotein (LDL) cholesterol, continuous

- High density lipoprotein (HDL) cholesterol, continuous
- Triglycerides, continuous
- Serum calcium, continuous
- Serum phosphate, continuous
- Serum bicarbonate, continuous
- Albuminuria, continuous
- Serum albumin, continuous

- *Medications*
  - Cardiovascular
    - ACE inhibitors
    - ARBs
    - Aldosterone receptor agonists (MRA)
    - Angiotensin receptor-neprilysin inhibitor (ARNI)
    - Loop diuretics
    - Beta blockers
    - Thiazide diuretics
    - Calcium channel blockers
    - Alpha blockers, e.g., clonidine and methyldopa
  - Glucose-lowering drugs
    - Metformin
    - Sulphonylurea
    - Dipeptidyl peptidase 4 inhibitors (DPP-4i)
    - Glucagon-like peptide-1 receptor agonist (GLP1-RA)
    - Insulin
    - Other oral anti-diabetes (OADs), (sulfonylureas, thiazolidinediones, biguanides, alpha-glucosidase inhibitors)
  - Lipid-lowering drugs
  - SGLT2 inhibitors
  - Antithrombotic/antiplatelet agents
  - Anticoagulants

- *Medical history/comorbidities*
  - Atherosclerotic cardiovascular disease (ASCVD)
    - Myocardial infarction (MI)
    - Unstable angina (UA)

    - Stable angina
    - Coronary revascularization
    - Stroke (ischemic or hemorrhagic)

- Heart failure
- Atrial fibrillation
- Peripheral arterial disease (PAD)
- Diabetes mellitus
    - Type 1 diabetes (T1D)
    - Type 2 diabetes (T2D)
    - Unclassified diabetes

- Hypertension
- Glomerulonephritis
- Hyperkalemia
- Hypertensive kidney failure
- Diabetic nephropathy
- Acute kidney injury (using diagnostics codes)
- Unspecified kidney disease
- Gout
- Poly cystic kidney disease
- Lupus
- Number of clinic visits in the year before the index date

4.4 Study Subgroups

The following study subgroups will be examined as part of this analysis:

- Age (in years)
- Gender
- Ethnicity
- T2D (Yes vs. No)
- Heart failure (Yes vs. No)
- T2D and heart failure
- Anemia (Yes vs. No)
- Hypertension (Yes vs. No)
- CKD stage 3a vs 3b
- UACR (<30, 30-300, >300 mg/g)

- Health care setting where eGFR is measured (inpatient vs outpatient)
- Etiology of CKD (Glomerulonephritis, Hypertensive kidney failure, Diabetic nephropathy, Acute kidney injury, Unspecified kidney disease)
- Established CVD (history of MI, stroke, CABG, PCI or unstable angina)

5. STATISTICAL ANALYSIS PLAN

5.1 Statistical Methods – General Aspects

This study is purely descriptive without any formal hypotheses. The main focus will be on quantifying the prevalence and consequences of undiagnosed CKD. Key assumptions include: our ability to define undiagnosed CKD subjects with only eGFR as an indicator of a future diagnosis of CKD by a physician, secondly, that absence of claims or health record data for CKD or other comorbid events indicate that the diagnosis of the condition never occurred, and finally, the timing of recorded CKD diagnosis in the database corresponds with the time when the first CKD diagnosis was made by the health care provider (assuming minimum lag in data recording).

Missing data for baseline measures will be assessed and addressed as a categorical variable with a level for missingness. All reported measures will be summarized in the study tables. Point estimates and their 95% C.I.s will be presented in the final analyses.

Data analysis will take place in a step-wise approach where the objectives pertinent to the describing the undiagnosed CKD patient cohort will be performed first and results will be reviewed by the study team before the analysis on the rest of the objectives take place.

5.2 Statistical Analyses

Baseline characteristics

Baseline characteristics will be described for the study population. Categorical variables will be summarized using patient counts with percentages, and continuous variables will be summarized using means with standard deviations (SDs), medians with interquartile ranges (IQRs), and minimum and maximum values.

Primary objective

- *Estimation of the prevalence of undiagnosed stage 3 CKD*

The prevalence numerator will include all patients who fulfill the undiagnosed stage 3 CKD case definition (see section 4.2) at any time during the observation period. The prevalence denominator will include all individuals meeting the inclusion criteria for this study (cohort 1). Prevalence will be estimated by dividing the numerator by the denominator, and 95% CIs around prevalence will be estimated using a binomial distribution. The denominator population should be the source of cases for the numerator. The prevalence of undiagnosed

stage 3 CKD (cohort 2) will be stratified according to patient subgroups of interest (section 4.4).

Sensitivity Analysis

To calculate the robustness of our undiagnosed CKD definition (identified through 2 eGFR measures and no ICD-9/10 CKD diagnosis codes, and OHDSI ICD-10 CN2016 is used in the CRDS database), we will conduct sensitivity analyses 1-2 that assess whether the lack of healthcare encounters, as originally defined, truly captures an undiagnosed population.

Sensitivity analysis for undiagnosed CKD includes ICD-9/10 diagnosis codes for stage 3 CKD or higher

Sensitivity analysis for undiagnosed CKD includes ICD-9/10 diagnosis codes for CKD based on a broader definition derived by Winkelmayer *et al.*, 2005[11] (section 8). We will conduct sensitivity analysis 3 to assess if we have included sufficient individuals with undiagnosed CKD in the database, as required by study inclusion criteria 2.

Sensitivity analysis for adjustment of inclusion criteria 2 from "at least 12 months of continuous presence in the database or registration in the data prior to the first qualifying eGFR (for data sources with information on enrollment)" to "at least one medical visit with diagnostic record within ≤12 months prior to the first qualifying eGFR". There are no changes in any other inclusion and exclusion criteria. Analyze undiagnosed CKD according to the primary study definitions using eGFR measures captured within a specific time period prior to diagnosis.

- *Time to CKD diagnosis*

For the cohort of patients with no CKD diagnosis code as of the study index date (cohort 2), the median (95% CI) time in days/months from the study index date to physician-diagnosed CKD (using the primary CKD study definition) will be calculated using the Kaplan-Meier method. Survival times will be censored as of the last day of follow-up for patients who remain undiagnosed by a physician. Time to physician diagnosis of CKD will be calculated overall and by patient characteristics and comorbidities. If possible, association between baseline characteristics and time to CKD diagnosis will be assessed using relevant regression models. The time to physician diagnosed stage 3 CKD or higher (sensitivity analysis #1) and the time to CKD using a broader definition (sensitivity analysis #2) (section 8) will also be calculated.

Secondary objectives

- *Trends over time*

Trends in the prevalence of undiagnosed CKD over time will be examined by calculating the prevalence during each calendar year of available data. Patients who have a second qualifying

eGFR measurement for stage 3 CKD (index date) during a given calendar year will contribute data toward the calculation of prevalence for that year. It is possible that some patients may contribute data as an undiagnosed stage 3 CKD case during one calendar year, and as a diagnosed stage 3 CKD case during a subsequent calendar year. However, once patients have a physician diagnosis of CKD, they cannot contribute data as an undiagnosed case during subsequent calendar years. It is also possible that some patients may have an initial diagnosis code for CKD that occurs <6 months following their qualifying eGFR, but in a subsequent calendar year. These patients should be classified as "diagnosed" during the year of their qualifying eGFR. In the same way, patients should be classified as "undiagnosed" during the year of their qualifying eGFR if their diagnosis code occurs >6 months following their qualifying eGFR in a subsequent calendar year.

- *Diagnosed versus undiagnosed CKD (baseline characteristics)*

Baseline characteristics will be summarized for diagnosed and undiagnosed CKD patients to compare relevant descriptive statistics and absolute standardized differences. A logistic regression model examining baseline factors associated with undiagnosed CKD will be conducted. The dependent variable is not having CKD diagnosed by a physician as previously described (e.g., presence vs. absence). Baseline characteristics as defined in section 4.3 will be included in the model. Parameter estimates along with odds ratios, 95% C.I.s and p-values will be reported.

The proportions of patients receiving treatment for CKD (ACEi, ARB and SGLT2i) within 12 months following the study index date will be assessed for the diagnosed and undiagnosed CKD groups. Additionally, patients prescribed CKD treatments in absence of a code for hypertension and/or heart failure will be assessed. These analyses will help assess the clinical applicability of our methods to detect undiagnosed patients e.g., whether a significant proportion of patients without codes for CKD are being treated for the condition with medications indicated for other uses, e.g., HTN, HF.

- *Quality indicators*

The proportion of patients achieving each of the quality indicators will be calculated for diagnosed and undiagnosed CKD patients. Estimates will be stratified by patient subgroups (section 4.4). Estimates will be calculated separately for all patients as well as the subset with complete follow-up data during the specified interval over which the quality measure is to be assessed (for data sources with information on enrollment).

5.3 Bias

5.3.1 Methods to Minimize Bias

The identification of patients with undiagnosed vs. diagnosed CKD relies on laboratory eGFR results, and the assumption that the absence of a CKD diagnosis code (e.g., ICD 9/10)

indicates that the patient is yet to be diagnosed with the condition by the physician. As this information is being inferred from EMR records, our assumptions may result in the misclassification of patients. The accuracy of our algorithm to identify patients with undiagnosed CKD is unknown. However, bias due to misclassification should be minimized, as our definition of CKD using eGFR results is based on an established clinical definition.[3] Additionally, for patients identified as “undiagnosed”, we will also examine whether therapies for CKD (e.g., ACEi, ARB) are administered in the 12 month period following the index date (2nd eGFR result between 30-<60) to understand the likelihood that the patient is actually undiagnosed e.g., a code for CKD does not appear in their medical records but they are receiving treatment for CKD.

5.3.2 Adjustment for Multiple Comparisons

Not applicable.

5.3.3 Study Limitations

Although large retrospective databases can be used to identify and track cohorts of patients representing real-world populations, the results of this study should be considered in the context of the biases that are inherent to this type of design.

This study is subject to the following limitations:

- Data collected in a real-world setting may lack information on specific covariates and laboratory investigations. We will, however, assess the level of covariate missingness. The potential impact of missingness on the outcome of interest will be investigated.
- Coding may be a limitation itself due to sensitivity and specificity, and caution is required when interpreting any clinical associations.
- The analysis will examine the proportion of undiagnosed CKD among those who are tested/screened for CKD only; this could have the effect of biasing the proportion of undiagnosed versus screening all individuals for CKD.
- There is a risk of misclassification (diagnosed vs. undiagnosed) if CKD diagnosis is made in clinical settings that do not contribute data to the selected database.
- The generalizability of the results of this study may not be applicable to CKD patients who differ from the study population or countries not included in the analysis.
- There may be unmeasured confounding that may have an impact on risk estimates and may differ between countries and settings

5.4 Interim Analyses (Optional)

Not applicable.

5.5 Sample Size and Power Calculations

If the prevalence of undiagnosed CKD is between 60% and 80%, then a sample size of 1,000 patients will be sufficient to estimate the prevalence rate with a margin of error of +/-3% in a 95% CI per institution/country (6% width of 95% CI).

According to phase I of REVEAL-CKD in China: in the feasibility assessment report the Prevalence and consequences of undiagnosed chronic kidney disease in China, the number of patients who met the inclusion and exclusion criteria in the Chinese Renal Disease Data System (CRDS) database is about 27,000 and the number of patients who met the inclusion and exclusion criteria of sensitivity analysis in the CRDS database is about 46,000[3].

6. STUDY CONDUCT AND REGULATORY DETAILS

6.1 Study Conduct

The study proposal will be reviewed by the Evidence Review Team (ERT) for approval. Upon approval, a statistical analysis plan (SAP) will be created to outline the statistical procedure/table shells etc. Relevant approvals will be obtained for data use where necessary (e.g. local ethics approval). Results will be presented as abstracts and manuscripts.

6.1.1 Procedures

Not applicable (no primary data collection).

6.1.2 Quality Control

Data management and analyses will be performed by a local vendor in China. Quality control of programming will be performed via double-checks of programming codes by an independent programmer.

The extent of missing data will be investigated, and concerns for bias resulting from missing data will be addressed (e.g., via sensitivity analyses), as deemed appropriate based on the variable.

6.2 Protection of Human Subjects

The observational study will be performed in accordance with ethical principles that are consistent with the Declaration of Helsinki, International Conference on Harmonisation (ICH) Good Clinical Practices (GCPs), Guidelines for Good Pharmacoepidemiology Practices (GPP), and the applicable legislation on Non-Interventional Studies and/or Observational Studies.

The Investigator will perform the observational study in accordance with the regulations and guidelines governing medical practice and ethics in the country of the observational study and in accordance with currently acceptable techniques and know-how.

6.2.1 Subject Informed Consent (Primary Data Collection Only)

Not applicable (no primary data collection).

6.2.2 Confidentiality of Study/Subject Data (Primary Data Collection Only)

This study does not involve any primary data collection. Data will be extracted from existing databases and stored in accordance with local regulations for data protection, to protect patient confidentiality. Results will be presented on an aggregate level, ensuring that no individual can be identified.

6.3 Collection and Reporting of Adverse Events/Adverse Drug Reactions

As this study is solely based on secondary data (no primary data collection will be performed), there is no requirement for adverse events to be recorded nor reported.

7. LIST OF REFERENCES

1. Bakris G. Prevalence and factors associated with undiagnosed chronic kidney disease in diabetes mellitus. *National Kidney Foundation 2019 Spring Clinical Meetings.* 2019.
2. Bello AK, Ronksley PE, Tangri N, et al. Quality of Chronic Kidney Disease Management in Canadian Primary Care. *JAMA Network Open.* 2019;2(9):e1910704-e1910704.
3. In house data, AstraZeneca. REVEAL-CKD: Prevalence and consequences of undiagnosed chronic kidney disease in China——Feasibility assessment report
4. The Renal Association. CKD stages. 2020; https://renal.org/information-resources/the-uk-eckd-guide/ckd-stages. Accessed 19 October 2020.
5. Centers for Disease Control and Prevention (CDC). National Chronic Kidney Disease Fact Sheet, 2017. 2017; https://www.cdc.gov/diabetes/pubs/pdf/kidney_Factsheet.pdf. Accessed 19 October 2020.
6. de Lusignan S, Tomson C, Harris K, van Vlymen J, Gallagher H. UK Prevalence of Chronic Kidney Disease for the Adult Population Is 6.76% Based on Two Creatinine Readings. *Nephron Clinical Practice.* 2012;120(No. 2):107.
7. Xie Y, Bowe B, Mokdad AH, et al. Analysis of the Global Burden of Disease study highlights the global, regional, and national trends of chronic kidney disease epidemiology from 1990 to 2016. *Kidney International.* 2018;94:567-581.
8. Gansevoort RT, Correa-Rotter R, Hemmelgarn BR, et al. Chronic kidney disease and cardiovascular risk: epidemiology, mechanisms, and prevention. *Lancet.* 2013;382(9889):339-352.
9. Mayo Clinic. Chronic kidney disease: Diagnosis & treatment. 2020; https://www.mayoclinic.org/diseases-conditions/chronic-kidney-disease/diagnosis-treatment/drc-20354527. Accessed 19 October 2020.
10. Go AS, Chertow GM, Fan D, McCulloch CE, Hsu CY. Chronic kidney disease and the risks of death, cardiovascular events, and hospitalization. *N Engl J Med.* 2004;351(13):1296-1305.
11. Winkelmayer WC, Schneeweiss S, Mogun H, Patrick AR, Avorn J, Solomon DH. Identification of individuals with CKD from Medicare claims data: a validation study. *American journal of kidney diseases : the official journal of the National Kidney Foundation.* 2005;46(2):225-232.

8. APPENDICES

Table A1. ICD-9/10 diagnosis coding definitions for identifying CKD

Description	ICD-9	ICD-10	ICD-10CN2016#
**Primary Study Definition**			
Chronic kidney disease, Stage (I)	585.1	N18, N18.1	N18, N18.801
Chronic kidney disease, Stage (II)	585.2	N18.2	N18.802
Chronic kidney disease, Stage (III)	585.3	N18.3, N18.30,N18.31,N18.32	N18.803
Chronic kidney disease, Stage (IV) (severe)	585.4	N18.4	N18.804
Chronic kidney disease, Stage V	585.5	N18.5	N18.001, N18.002(D63.8*)
End stage renal disease	585.6	N18.6	N18.0, N18.805(J99.8*), N18.806(G94.8*), N18.807(G63.8*), N18.808(I32.8*), N18.809(I43.8*), N19.x01
Chronic kidney disease, unspecified	585.9	N18.9	N18.9
Hypertensive CKD	403.x, 404.x	I12.x, I13.x	I12, I13
Diabetes with renal manifestation	250.4x	E10.2.x, E11.2x, E13.2x, E14.2	E10.2, E11.2, E12.2, E13.2, E14.2,
Disorders from impaired renal function	588.x	N25	N25
**Sensitivity Analyses #1**			
Chronic kidney disease, Stage (III)	585.3	N18.3, N18.30, N18.31, N18.32	N18.803
Chronic kidney disease, Stage (IV) (severe)	585.4	N18.4	N18.804
Chronic kidney disease, Stage V	585.5	N18.5	N18.001, N18.002(D63.8*)
End stage renal disease	585.6	N18.6	N18.000, N18.805(J99.8*), N18.806(G94.8*), N18.807(G63.8*), N18.808(I32.8*), N18.809(I43.8*), N19.x01
**Sensitivity Analyses #2***			
Chronic renal insufficiency	582, 583, 585, 586, 587	N03, N05, N18, N19, N26.9	N03, N03.0, N03.1, N03.2, N03.3, N03.4, N03.5, N03.6, N03.7, N03.8, N03.9, N05, N05.0, N05.1, N05.2, N05.3, N05.4, N05.5, N05.6, N05.7, N05.8, N05.9, N18, N18.801, N18.802, N18.803, N18.804, N18.001, N18.002(D63.8*), N18.0,

Description	ICD-9	ICD-10	ICD-10CN2016#
			N18.9, N19, N26.x01, N26.x02, N26.x00, N11.8, N11.9

Sensitivity Analyses #2 (continued)			
Hypertensive nephropathy	403.xx, 404.xx	I12, I13	I12, I13
Acute renal failure	572.4, 580.xx, 584.xx, 580.0, 580.4, 580.89, 580.9, 582.4, 791.2, 791.3	K76.7, N00.3, N01.3, N08, N00.8, N00.9, N17, N03.8, R82.3, R82.1	K76.7, N00.3, N00.8, N00.9, N01.3, N03.8, N17, N17.0, N17.1, N17.2, N17.8, N17.9, R82.1, R82.3, N10.x00, N10.x01
Miscellaneous	274.10, 440.1, 442.1, 453.3, 581.xx, 593.xx, 753.0, 753.3, 866.00, 866.01, 866.1	M10.3, I70.1, I72.2, I82.3, N04, N02.2, N08, N28.81, N28.83, N28.1, N13.5, N13.8, N13.4, N13.7, N28.80, N28.89, N28.82, N28.9, R80.2, Q60.2, Q60.5, Q63.0-Q63.8, S37.009, S37.019, S37.029, S37.039, S37.049, S37.059, S37.069, S31.001,	I70.1, I72.2, I82.3, M10.3, N02.2, N04, N04.0, N04.1, N04.2, N04.3, N04.4, N04.5, N04.6, N04.7, N04.8, N04.9, N08*, N13.4, N13.5, N13.7, N13.700, N13.701, N13.8, N28.1, N28.8, N28.807, N28.821, N28.812, N28.800, N28.9, Q60.2, Q60.5, Q63, Q63.0, Q63.1, Q63.2, Q63.3, Q63.8, Q63.9, R80, S31.0, S31.000, S37.0, S37.000, S37.001, S37.002, S37.003, S37.004, S37.010, S37.011, N07, N06, N12, N14, N15, N16*, Q61, N25

* **Adapted from Winkelmayer et al. (2005)**

# Only OHDSI ICD-10CN2016 is used in CRDS database and ICD-9/10 is listed for reference.

9. SIGNATURE

ASTRAZENECA SIGNATURE(S)

REVEAL-CKD – Prevalence of undiagnosed chronic kidney disease

This Observational Study Protocol has been subjected to an internal AstraZeneca review
I agree to the terms of this Study protocol.

AstraZeneca representative

Signature ______________________ ______________

[redacted]

[redacted] ______________

AstraZeneca representative

____

Signature ______________

[redacted]

INVESTIGATOR SIGNATURES

REVEAL-CKD: Prevalence and consequences of undiagnosed chronic kidney disease

A multinational observational study to determine the prevalence and consequences of undiagnosed chronic kidney disease

This Study Protocol has been subjected to internal AstraZeneca review. I agree to the terms of this study protocol. I will conduct the study according to the procedures specified herein, and according to the local regulations.

Investigator

______________________________ ________________

[redacted]

This document contains confidential information, which should not be copied, referred to, released or published without written approval from AstraZeneca. Investigators are cautioned that the information in this protocol may be subject to change and revision.
