# Supplementary material for: Characteristics of patients with undiagnosed stage 3 chronic kidney disease: results from an observational study (REVEAL-CKD) in China
Source: Lancet Reg Health West Pac. 2025 Jan 4;54:101275. doi: 10.1016/j.lanwpc.2024.101275 (PMC11758424; doi:10.1016/j.lanwpc.2024.101275)
Supplement: Supplementary Materials [file mmc3.docx]

**Characteristics of patients with undiagnosed stage 3 chronic kidney disease: results from the REVEAL-CKD study in China**

**Table of Contents**

[Supplementary Method 2](#_Toc182848084)

[Supplementary Table S1. Patient distribution in 20 contributing hospitals from CRDS 3](#_Toc182848085)

[Supplementary Table S2. ICD codes for the diagnosis status of CKD 4](#_Toc182848086)

[Supplementary Table S3. ICD codes for baseline comorbidities 6](#_Toc182848087)

[Supplementary Table S4. ATC codes for baseline medication use 9](#_Toc182848088)

[Supplementary Table S5. Proportion of patients with missing data for laboratory characteristics 10](#_Toc182848089)

[Supplementary Table S6. Multivariate analysis of factors associated with undiagnosed CKD in sensitivity analysis 3 11](#_Toc182848090)

[Supplementary Table S7. CKD management quality indicators within 180 days before and after CKD diagnosis 12](#_Toc182848091)

[Supplementary Fig. S1. Kaplan-Meier estimates of time to CKD diagnosis among the undiagnosed patients (a) overall; (b) by age group; (c) by sex; (d) by CKD stage; and (e) by diabetes status 13](#_Toc182848092)

# Supplementary Method

$$\log\left( {eGFR}_{ijk} \right)=\beta_{0}+\beta_{1}t_{ij}+\beta_{2}{diagnosis}_{i}+\beta_{3}{(diagnosis}_{i}\times t_{ij})+\beta_{4}{base.egfr}_{i}+\beta_{5}{Comedications}_{i}+\beta_{6}{Comorbidities}_{i}+\beta_{7}{hosp}_{k}+b_{0j}+b_{1j}t_{ij}+\mu_{0k}+\epsilon$$

$b_{0j}$ represents the deviation of the intercept for patient j from the overall intercept $\beta_{0}$​. It accounts for individual differences in baseline log(eGFR) that are not explained by the fixed effects

$b_{1j}$ represents the deviation of the time slope for patient j from the overall time slope $\beta_{1}$​. It accounts for individual differences in the rate of change of log(eGFR) over time that are not explained by the fixed effects.

$\mu_{0k}$ represents the deviation of the intercept for hospital k from the overall intercept $\beta_{0}$​. It accounts for differences in baseline log(eGFR) across different hospitals.

# Supplementary Table S1. Patient distribution in 20 contributing hospitals from CRDS

| **Region** | **Hospital Number** | **Number of patients in different hospital, n (%)** |
| --- | --- | --- |
| Eastern China (n=7) | 1 | 337 (1·0) |
|  | 2 | 1067 (3·0) |
|  | 3 | 1956 (5·6) |
|  | 4 | 2092 (5·9) |
|  | 5 | 2747 (7·8) |
|  | 6 | 2043 (5·8) |
|  | 7 | 0 (0)^a^ |
| Northern China (n=1) | 8 | 113 (0·3) |
| Northwestern China (n=1) | 9 | 454 (1·3) |
| Southern China (n=9) | 10 | 2110 (6·0) |
|  | 11 | 3994 (11·3) |
|  | 12 | 585 (1·7) |
|  | 13 | 6111 (17·3) |
|  | 14 | 985 (2·8) |
|  | 15 | 4662 (13·2) |
|  | 16 | 824 (2·3) |
|  | 17 | 3886 (11·0) |
|  | 18 | 469 (1·3) |
| Southwestern China (n=2) | 19 | 650 (1·8) |
|  | 20 | 137 (0·4) |

^a^Hospital 7 is a children’s hospital, with no patients aged ≥18 years included.

# Supplementary Table S2. ICD codes for the diagnosis status of CKD

| **Description** | **ICD-9** | **ICD-10** | **ICD-10CN2016^a^** |
| --- | --- | --- | --- |
| **ICD codes for CKD in primary analysis** | | | |
| Chronic kidney disease | 585.9 | N18 | N18 |
| Chronic kidney disease, Stage 1 | 585.1 | N18.1 | N18.801 |
| Chronic kidney disease, Stage 2 | 585.2 | N18.2 | N18.802 |
| Chronic kidney disease, Stage 3 | 585.3 | N18.3, N18.30, N18.31, N18.32 | N18.803 |
| Chronic kidney disease, Stage 4 (severe) | 585.4 | N18.4 | N18.804 |
| Chronic kidney disease, Stage 5 | 585.5 | N18.5 | N18.001, N18.002(D63.8*) |
| End stage renal disease | 585.6 | N18.6 | N18.0, N18.805(J99.8*), N18.806(G94.8*), N18.807(G63.8*), N18.808(I32.8*), N18.809(I43.8*), N19.x01 |
| Chronic kidney disease, unspecified | 585.9 | N18.9 | N18.9 |
| Hypertensive CKD | 403.x, 404.x | I12.x, I13.x | I12, I13 |
| Diabetes with renal manifestation | 250.4x | E10.2.x, E11.2x, E13.2x, E14.2 | E10.2, E11.2, E12.2, E13.2, E14.2 |
| Disorders from impaired renal function | 588.x | N25 | N25 |
| **ICD codes for stage 3 or higher CKD in sensitivity analysis 1** | | | |
| Chronic kidney disease, Stage (III) | 585.3 | N18.3, N18.30, N18.31, N18.32 | N18.803 |
| Chronic kidney disease, Stage (IV) (severe) | 585.4 | N18.4 | N18.804 |
| Chronic kidney disease, Stage V | 585.5 | N18.5 | N18.001, N18.002(D63.8*) |
| End stage renal disease | 585.6 | N18.6 | N18.000, N18.805(J99.8*), N18.806(G94.8*), N18.807(G63.8*), N18.808(I32.8*), N18.809(I43.8*), N19.x01 |
| **ICD codes for a broader definition of CKD in sensitivity analysis 2^b^** | | | |
| Chronic renal insufficiency | 582, 583, 585, 586, 587 | N03, N05, N18, N19, N26.9 | N03, N03.0, N03.1, N03.2, N03.3, N03.4, N03.5, N03.6, N03.7, N03.8, N03.9, N05, N05.0, N05.1, N05.2, N05.3, N05.4, N05.5, N05.6, N05.7, N05.8, N05.9, N18, N18.801, N18.802, N18.803, N18.804, N18.001, N18.002(D63.8*), N18.0, N18.9, N19, N26.x01, N26.x02, N26.x00, N11.8, N11.9 |
| Hypertensive nephropathy | 403.xx, 404.xx | I12, I13 | I12, I13 |
| Acute renal failure | 572.4, 580.xx, 584.xx, 580.0, 580.4, 580.89, 580.9, 582.4, 791.2, 791.3 | K76.7, N00.3, N01.3, N08, N00.8, N00.9, N17, N03.8, R82.3, R82.1 | K76.7, N00.3, N00.8, N00.9, N01.3, N03.8, N17, N17.0, N17.1, N17.2, N17.8, N17.9, R82.1, R82.3, N10.x00, N10.x01 |
| Miscellaneous | 274.10, 440.1, 442.1, 453.3, 581.xx, 593.xx, 753.0, 753.3, 866.00, 866.01, 866.1 | M10.3, I70.1, I72.2, I82.3, N04, N02.2, N08, N28.81, N28.83, N28.1, N13.5, N13.8, N13.4, N13.7, N28.80, N28.89, N28.82, N28.9, R80.2, Q60.2, Q60.5, Q63.0-Q63.8, S37.009, S37.019, S37.029, S37.039, S37.049, S37.059, S37.069, S31.001, | I70.1, I72.2, I82.3, M10.3, N02.2, N04, N04.0, N04.1, N04.2, N04.3, N04.4, N04.5, N04.6, N04.7, N04.8, N04.9, N08*, N13.4, N13.5, N13.7, N13.700, N13.701, N13.8, N28.1, N28.8, N28.807, N28.821, N28.812, N28.800, N28.9, Q60.2, Q60.5, Q63, Q63.0, Q63.1, Q63.2, Q63.3, Q63.8, Q63.9, R80, S31.0, S31.000, S37.0, S37.000, S37.001, S37.002, S37.003, S37.004, S37.010, S37.011, N07, N06, N12, N14, N15, N16*, Q61, N25 |

^a^The ICD coding system varied by country and only ICD-10CN2016 codes were available in CRDS. ICD-9/10 codes are listed for reference. ^b^Adapted from Winkelmayer WC, Schneeweiss S, Mogun H, et al. Identification of individuals with CKD from medicare claims data: a validation study. Am J of Kidney Dis 2005;46:225–232.

CKD, chronic kidney disease; CRDS, China Renal Data System; ICD, International Classification of Diseases

# Supplementary Table S3. ICD codes for baseline comorbidities

| **Description** | **ICD-10** | **ICD-10 CN2016^a^** |
| --- | --- | --- |
|  | | |
| Chronic kidney disease, Stage 1 | N18, N18.1 | N18, N18.801 |
| Chronic kidney disease, Stage 2 | N18.2 | N18.802 |
| Chronic kidney disease, Stage 3 | N18.3, N18.30, N18.31, N18.32 | N18.803 |
| Chronic kidney disease, Stage 4 (severe) | N18.4 | N18.804 |
| Chronic kidney disease, Stage 5 | N18.5 | N18.001, N18.002(D63.8*) |
| End stage renal disease | N18.6 | N18.0, N18.805(J99.8*), N18.806(G94.8*), N18.807(G63.8*), N18.808(I32.8*), N18.809(I43.8*), N19.x01 |
| Chronic kidney disease, unspecified | N18.9 | N18.9 |
| Hypertensive CKD | I12.x, I13.x | I12, I13 |
| Diabetes with renal manifestation | E10.2.x, E11.2x, E13.2x, E14.2 | E10.2, E11.2, E12.2, E13.2, E14.2 |
| Disorders from impaired renal function | N25.x | N25 |
|  | | |
| Chronic kidney disease, Stage (III) | N18.3, N18.30, N18.31, N18.32 | N18.803 |
| Chronic kidney disease, Stage (IV) (severe) | N18.4 | N18.804 |
| Chronic kidney disease, Stage V | N18.5 | N18.001, N18.002(D63.8*) |
| End stage renal disease | N18.6 | N18.000, N18.805(J99.8*), N18.806(G94.8*), N18.807(G63.8*), N18.808(I32.8*), N18.809(I43.8*), N19.x01 |
|  | | |
| Chronic renal insufficiency | N03, N05, N18, N19, N26.9 | N03, N03.0, N03.1, N03.2, N03.3, N03.4, N03.5, N03.6, N03.7, N03.8, N03.9, N05, N05.0, N05.1, N05.2, N05.3, N05.4, N05.5, N05.6, N05.7, N05.8, N05.9, N18, N18.801, N18.802, N18.803, N18.804, N18.001, N18.002(D63.8*), N18.0, N18.9, N19, N26.x01, N26.x02, N26.x00, N11.8, N11.9 |
| Hypertensive nephropathy | I12, I13 | I12, I13 |
| Acute renal failure | K76.7, N00.3, N01.3,  N08, N00.8, N00.9, N17,  N03.8, R82.3, R82.1 | K76.7, N00.3, N00.8, N00.9, N01.3, N03.8, N17, N17.0, N17.1, N17.2, N17.8, N17.9, R82.1, R82.3, N10.x00, N10.x01 |
| Miscellaneous | M10.3, I70.1, I72.2, I82.3,  N04, N02.2, N08, N28.81,  N28.83, N28.1, N13.5,  N13.8, N13.4, N13.7, N28.80, N28.89, N28.82, N28.9, R80.2, Q60.2,  Q60.5, Q63.0-Q63.8, S37.009, S37.019,  S37.029, S37.039,  S37.049, S37.059,  S37.069, S31.001, | I70.1, I72.2, I82.3, M10.3, N02.2, N04, N04.0, N04.1, N04.2, N04.3, N04.4, N04.5, N04.6, N04.7, N04.8, N04.9, N08*, N13.4, N13.5, N13.7, N13.700, N13.701, N13.8, N28.1, N28.8, N28.807, N28.821, N28.812, N28.800, N28.9, Q60.2, Q60.5, Q63, Q63.0, Q63.1, Q63.2, Q63.3, Q63.8, Q63.9, R80, S31.0, S31.000, S37.0, S37.000, S37.001, S37.002, S37.003, S37.004, S37.010, S37.011, N07, N06, N12, N14, N15, N16*, Q61, N25 |
|  |  |  |
| MI |  | I21(Includes 3-digit category codes, 4-digit subcategory codes, and 6-digit primary disease codes) |
| Old MI |  | I25.2, I21.3, I21.4, I21.9(Includes 4-digit subcategory codes, and 6-digit primary disease codes) |
| Unstable angina |  | I20.0(Includes 4-digit subcategory codes, and 6-digit primary disease codes) |
| Stable angina |  | I20.1，I20.8，I20.9(Includes 4-digit subcategory codes, and 6-digit primary disease codes) |
| Stroke |  | I60, I61, I62, I63(Includes 3-digit category codes, 4-digit subcategory codes, and 6-digit primary disease codes), O22.5, O87.3(Includes 4-digit subcategory codes, and 6-digit primary disease codes) |
| Stroke TIA |  | G45 |
| Heart failure |  | I11.0, I13.0, I13.2(Includes 4-digit subcategory codes, and 6-digit primary disease codes), I50(Includes 3-digit category codes, 4-digit subcategory codes, and 6-digit primary disease codes) |
| Arrhythmia atrial fibrillation |  | I48(Includes 3-digit category codes, 4-digit subcategory codes, and 6-digit primary disease codes) |
| Pad peripheral arterial disease |  | I70.2, I70.8, I70.9, I74.2, I74.3, I74.4,I74.5(Includes 4-digit subcategory codes, and 6-digit primary disease codes) |
| Type 1 diabetes |  | E10(Includes 3-digit category codes, 4-digit subcategory codes, and 6-digit primary disease codes), O24.0(Includes 4-digit subcategory codes, and 6-digit primary disease codes) |
| Type 2 diabetes |  | E11, E13 (Includes 3-digit category codes, 4-digit subcategory codes, and 6-digit primary disease codes), O24.1(Includes 4-digit subcategory codes, and 6-digit primary disease codes) |
| Unclassified diabetes |  | E14 (Includes 3-digit category codes, 4-digit subcategory codes, and 6-digit primary disease codes) |
| Hypertension |  | I10, I11, I12, I13, I15, O10, O16 (Includes 3-digit category codes, 4-digit subcategory codes, and 6-digit primary disease codes), P29.2 (Includes 4-digit subcategory codes, and 6-digit primary disease codes) |
| Chronic nephritic syndrome |  | N03(Includes 3-digit category codes, 4-digit subcategory codes, and 6-digit primary disease codes) |
| Hyperkalaemia |  | E87.5(Includes 4-digit subcategory codes, and 6-digit primary disease codes) |
| Hypertensive kidney failure |  | I12(Includes 3-digit category codes, 4-digit subcategory codes, and 6-digit primary disease codes), I13.1, I13.2(Includes 4-digit subcategory codes, and 6-digit primary disease codes) |
| Diabetic nephropathy |  | E10.2, E11.2, E13.2(Includes 4-digit subcategory codes, and 6-digit primary disease codes), N08.3* |
| Acute kidney injury |  | D59.3, K76.7(Includes 4-digit subcategory codes, and 6-digit primary disease codes), N17(Includes 3-digit category codes, 4-digit subcategory codes, and 6-digit primary disease codes) |
| Unspecified kidney disease |  | N18.9(Includes 4-digit subcategory codes, and 6-digit primary disease codes) |
| Gout |  | M10(Includes 3-digit category codes, 4-digit subcategory codes, and 6-digit primary disease codes) |
| Polycystic kidney disease |  | Q61.1, Q61.2, Q61.3(Includes 4-digit subcategory codes, and 6-digit primary disease codes) |
| Lupus nephritis |  | M32(Includes 3-digit category codes, 4-digit subcategory codes, and 6-digit primary disease codes) |

^a^The ICD coding system varied by country and only ICD-10CN2016 codes were available in CRDS. ICD-10 codes are listed for reference.

CKD, chronic kidney disease; ICD, International Classification of Diseases; TIA, transient ischemic attack; MI, myocardial infarction

# Supplementary Table S4. ATC codes for baseline medication use

| **Description** |  | **ATC code** | **Notes** |
| --- | --- | --- | --- |
| Cardiovascular | ACE inhibitors | C09AA | Plain |
|  | ARBs | C09CA | Plain |
|  | Aldosterone receptor agonists | C03DA | Code of antagonists |
|  | ARNI | C09DX04 | Plain |
|  | Diuretics | C03 | code of diuretics |
|  | Beta blockers | C07A |  |
|  | Thiazide diuretics | C03AA | Plain |
|  | Calcium channel blockers | C08 |  |
|  | Alpha blockers, e.g., clonidine and methyldopa | C02A/C02C | Both categories have this type of medication |
| Glucose-lowering drugs | Metformin | A10BA02 |  |
|  | Sulphonylurea | A10BB |  |
|  | DPP-4 inhibitors | A10BH |  |
|  | GLP1-RA | A10BJ |  |
|  | Insulin | A10A |  |
|  | Other oral anti-diabetes (sulfonylureas, thiazolidinediones, biguanides, alpha-glucosidase inhibitors) | sulfonylureas: A10BB;  thiazolidinediones: A10BG;  biguanides: A10BA;  alpha-glucosidase inhibitors: A10BF |  |
| Lipid-lowering drugs |  | C10 | Plain: C10A; combination: C10B |
| SGLT2 inhibitors |  | A10BK |  |
| Antithrombotic/ antiplatelet agents |  | B01A/B01AC |  |
| Anticoagulants |  | B01A |  |

ACE, angiotensin-converting enzyme; ARB, angiotensin receptor blocker; ARNI, angiotensin receptor-neprilysin inhibitor; ATC, Anatomical Therapeutic Chemical; DPP-4, dipeptidyl-peptidase 4; GLP1-RA, glucagon-like peptide-1 receptor agonist; SGLT2, sodium-glucose cotransporter-2

# Supplementary Table S5. Proportion of patients with missing data for laboratory characteristics

| **Characteristic, n (%)** | **Overall**  **(N=35222)** | **Undiagnosed  stage 3 CKD  (n=25214)** | **Diagnosed  stage 3 CKD  (n=10008)** |
| --- | --- | --- | --- |
| Serum uric acid | 2731 (7·7%) | 2196 (8·7%) | 535 (5·3%) |
| Total cholesterol | 6902 (19·6%) | 5177 (20·5%) | 1725 (17·2%) |
| LDL cholesterol | 5459 (15·5%) | 3868 (15·3%) | 1591 (15·9%) |
| Serum albumin | 4159 (11·8%) | 3217 (12·8%) | 942 (9·4%) |

CKD, chronic kidney disease; LDL, low-density lipoprotein

# Supplementary Table S6. Multivariate analysis of factors associated with undiagnosed CKD in sensitivity analysis 3

| **Characteristics** | **OR (95% CI)** | **P value^a^** |
| --- | --- | --- |
| **Patient characteristics** |  |  |
| Age group (vs. <45), years |  |  |
| 45–<65 | 1·12 (0·97–1·28) | 0·116 |
| 65–<75 | 1·82 (1·58–2·09) | <0·001 |
| ≥75 | 2·53 (2·19–2·91) | <0·001 |
| Male (vs. female) | 0·54 (0·51–0·58) | <0·001 |
| With nephrology visit (vs. without) | 0·30 (0·28–0·33) | <0·001 |
| CKD stage 3B (vs. 3A) | 0·38 (0·35–0·40) | <0·001 |
| Potassium (per 1-SD increment) | 0·92 (0·89–0·95) | <0·001 |
| LDL cholesterol (per 1-SD increment) | 1·04 (1·00–1·08) | 0·039 |
| Albumin (per 1-SD increment) | 1·14 (1·10–1·18) | <0·001 |
| Serum uric acid (per 1-SD increment) | 0·93 (0·90–0·95) | <0·001 |
| Haemoglobin (per 10 g/l increment) | 1·18 (1·14–1·22) | <0·001 |
| Urine protein^b^ (vs. A1) |  |  |
| A2 | 0·72 (0·66–0·79) | <0·001 |
| A3 | 0·56 (0·51–0·63) | <0·001 |
| A4 | 0·55 (0·48–0·63) | <0·001 |
| Unknown | 1·24 (1·16–1·33) | <0·001 |
| **Comorbidities (vs. no history)** |  |  |
| Established CVD^c^ | 0·85 (0·79–0·9) | <0·001 |
| Heart failure | 0·78 (0·73–0·84) | <0·001 |
| Hypertension | 0·70 (0·66–0·75) | <0·001 |
| Any diabetes^d^ | 0·51 (0·48–0·55) | <0·001 |
| Chronic nephritic syndrome | 0·65 (0·57–0·74) | <0·001 |
| **Use of medications (vs. no history)** |  |  |
| RASi | 0·93 (0·87–1·00) | 0·036 |
| Other antihypertensive medications | 0·84 (0·79–0·91) | <0·001 |
| Lipid-lowering agents | 0·83 (0·78–0·89) | <0·001 |
| Antidiabetic agents | 0·93 (0·85–1·02) | 0·107 |

^a^Logistic regression model. ^b^Combination of UACR, 24-hour urine protein excretion, 24-hour urine albumin excretion, and semi-quantitative urine protein measurement, and categorised into A1 (UACR <30 mg/g, 24-hour urine microalbumin <30 mg/24h, 24-hour urine protein <150 mg/24h, or urine dipstick reading of negative or trace), A2 (UACR 30–300 mg/g, 24-hour urine microalbumin 30–300 mg/24h, 24-hour urine protein 150–500 mg/24h, or urine dipstick reading of 1+), A3 (UACR >300–2200 mg/g, 24-hour urine microalbumin >300–2200 mg/24h, 24-hour urine protein >500–3500 mg/24h, or urine dipstick reading of 2+), and A4 (UACR >2200 mg/g, 24-hour urine microalbumin >2200 mg/24h, 24-hour urine protein >3500 mg/24h, or urine dipstick reading of ≥3+). ^c^Included history of myocardial infarction, stroke, coronary artery bypass graft, percutaneous coronary intervention, or unstable angina. ^d^Included type 1, type 2, and other subtypes of diabetes.

CI, confidence interval; CKD, chronic kidney disease; CVD, cardiovascular disease; LDL, low-density lipoprotein; OR, odds ratio; RASi, renin-angiotensin system inhibitor; SD, standard deviation

# Supplementary Table S7. CKD management quality indicators within 180 days before and after CKD diagnosis

| **Characteristics** | **Before diagnosis (n=10008)** | **After diagnosis**  **(n=10008)** |
| --- | --- | --- |
| Monitoring rates (95% CI)^a^, person-years |  |  |
| Systolic blood pressure | 7·9 (7·7–8·1) | 8·1 (7·9–8·3) |
| Creatinine | 3·4 (3·3–3·5) | 3·3 (3·2–3·4) |
| Urine protein^b^ | 2·1 (2·1–2·1) | 2·2 (2·1–2·3) |
| LDL cholesterol | 1·0 (1·0–1·0) | 0·9 (0·8–1·0) |
| HbA1c | 0·5 (0·5–0·5) | 0·3 (0·3–0·3) |
| Serum uric acid | 2·5 (2·4–2·6) | 2·5 (2·4–2·6) |
| Serum potassium | 3·4 (3·3–3·5) | 3·4 (3·3–3·5) |
| Rate of meeting CKD management targets,  person-years (%) |  |  |
| Systolic blood pressure <120 mmHg | 2·0 (24·8) | 2·0 (25·3) |
| Urine protein <A2 | 0·8 (38·1) | 0·8 (36·4) |
| HbA1c <6·5% | 0·2 (48·7) | 0·1 (46·8) |
| LDL cholesterol <1·8 mmol/l | 0·2 (23·2) | 0·2 (26·6) |
| Serum uric acid <420 µmol/l | 1·1 (47·6) | 1·2 (49·6) |
| Serum potassium <5 mmol/l | 3·2 (91·1) | 3·1 (89·4) |

The analysis was performed in the 10008 patients receiving a CKD diagnostic code during the baseline period. ^a^Monitoring rates were collected in both inpatient and outpatient settings. ^b^Combination of UACR, 24-hour urine protein excretion, 24-hour urine albumin excretion, and semi-quantitative urine protein measurement and categorised into A1 (UACR <30 mg/g, 24-hour urine microalbumin <30 mg/24h, 24-hour urine protein <150 mg/24h, or urine dipstick reading of negative or trace), A2 (UACR 30–300 mg/g, 24-hour urine microalbumin 30–300 mg/24h, 24-hour urine protein 150–500 mg/24h, or urine dipstick reading of 1+), A3 (UACR >300–2200 mg/g, 24-hour urine microalbumin >300–2200 mg/24h, 24-hour urine protein >500–3500 mg/24h, or urine dipstick reading of 2+) and A4 (UACR >2200 mg/g, 24-hour urine microalbumin >2200 mg/24h, 24-hour urine protein >3500 mg/24h, or urine dipstick reading of ≥3+).

CI, confidence interval; CKD, chronic kidney disease; HbA1c, glycated haemoglobin; LDL, low-density lipoprotein; UACR, urine albumin-creatinine ratio


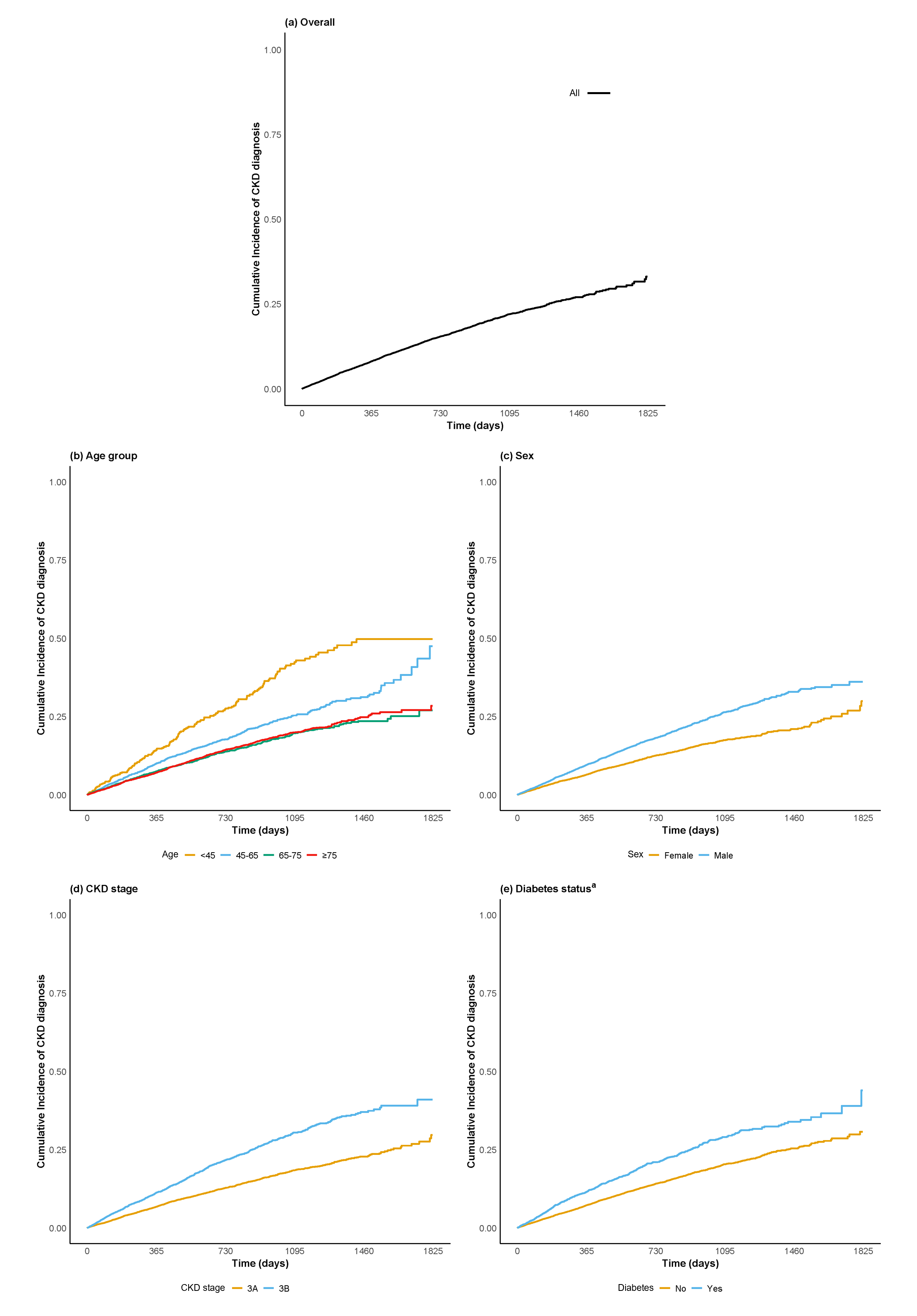


# **Supplementary Fig. S1**. Kaplan-Meier estimates of time to CKD diagnosis among the undiagnosed patients (a) overall; (b) by age group; (c) by sex; (d) by CKD stage; and (e) by diabetes status

The Kaplan-Meier estimates were calculated for all patients with undiagnosed CKD, regardless of whether they received a diagnosis during the follow-up period, and were presented starting from six months post index date. ^a^Included any subtype of diabetes. CKD, chronic kidney disease
