# Supplementary material for: Characteristics of patients with undiagnosed stage 3 chronic kidney disease: results from an observational study (REVEAL-CKD) in China
Source: Lancet Reg Health West Pac. 2025 Jan 4;54:101275. doi: 10.1016/j.lanwpc.2024.101275 (PMC11758424; doi:10.1016/j.lanwpc.2024.101275)
Supplement: Statement of Role of Medical Writer [file mmc1.pdf]

Prof Jie Cai
Editor-in-Chief
*The Lancet Regional Health — Western Pacific*

20 November 2024

**Statement of Role of Medical Writer**

Manuscript Title: **<u>Characteristics of patients with undiagnosed stage 3 chronic kidney disease: results from the REVEAL-CKD study in China</u>**

Dear Prof Cai,

I, Fan Fan Hou, the corresponding author of the above-named manuscript, acknowledge Xiaowei Ning and Ruilin Chen from Costello Medical Singapore (sponsored by AstraZeneca China) for writing assistance and editorial support based on the authors' input and direction.

Yours faithfully,

< Fan Fan Hou >

Fan Fan Hou, MD, PhD
Division of Nephrology,
Nanfang Hospital, Southern Medical University,
1838 North Guangzhou Ave,
Guangzhou 510515, China
Email: ffhouguangzhou@163.com
